# NFAT single-deficient murine T cells reduce the risk of aGvHD while controlling cytomegalovirus infection

**DOI:** 10.1016/j.isci.2025.111937

**Published:** 2025-02-01

**Authors:** Nadine Hundhausen, Snigdha Majumder, Yin Xiao, Sigrun S. Haeusl, Helen Goehler, Rishav Seal, Cristina M. Chiarolla, Andreas Rosenwald, Matthias Eyrich, Luka Cicin-Sain, Friederike Berberich-Siebelt

**Affiliations:** 1 Institute of Pathology, Julius-Maximilians-University Würzburg, Würzburg, Germany; 2 Comprehensive Cancer Centre Mainfranken, Julius-Maximilians-University Würzburg, Würzburg, Germany; 3 Department of Pediatrics, University Hospital Würzburg, Würzburg, Germany; 4 Department of Viral Immunology, Helmholtz Centre for Infection Research, Braunschweig, Germany; 5 Centre for Individualized Infection Medicine, a Joint Venture of Helmholtz Centre for Infection Research and Medical School Hannover, Hannover, Germany

**Keywords:** Natural sciences, Biological sciences, Immunology, Microbiology, Virology

## Abstract

NFAT is a family of transcription factors whose activation is inhibited by calcineurin inhibitors (CNIs). In allogeneic hematopoietic stem cell transplantation (allo-HCT), CNIs are employed to prevent and treat graft-versus-host disease (GvHD). Unfortunately, control of cytomegalovirus (CMV), which exacerbates clinical outcomes, is simultaneously lost. Since single NFAT deficiency in T cells ameliorates GvHD in our major mismatch model, we investigated whether protection is maintained during CMV infection. Reassuringly, NFAT-deficient T cells still improved GvHD upon acute CMV infection and after allo-HCT in latently CMV-infected mice, showing reduced proinflammatory and cytotoxic potential. In sharp contrast, CMV-specific NFAT-deficient CD8^+^ inflated memory T cells expanded more and with higher levels of interferon gamma (IFN-γ) and GzmB expression, effectively controlling CMV. Notably, NFAT-deficient inflated memory T cells could migrate to non-lymphoid tissues and fight CMV. Therefore, CMV infection does not interfere with the protective effect of NFAT inhibition to attenuate GvHD while allowing an anti-CMV response.

## Highlights

• NFAT-deficient co-transplanted T cells ameliorate GvHD despite MCMV infection
• NFAT-deficient T cells express less pro-inflammatory and cytotoxic molecules
• NFAT-deficient MCMV-responsive CD8^+^ T cells expand with heightened IFN-γ and GzmB
• NFAT-deficient T cells control MCMV better than WT T cells

## Graphical abstract

**Figure:**
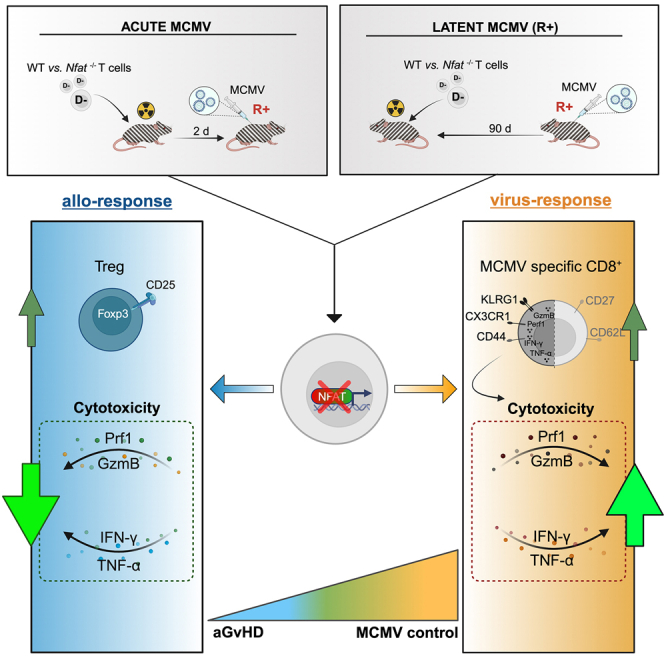

## Teaser

Immunology; Virology

## Introduction

Although associated with high risks, allogeneic hematopoietic stem cell transplantation (allo-HCT) is a curative treatment option for many hematologic malignancies. Despite intensive conditioning of the patients with chemotherapy and/or irradiation, malignant cells may persist and potentially cause tumor relapse. In addition to conditioning, donor-derived T cells find and eliminate the residual malignant cells, a reaction known as graft-versus-leukemia (GvL) effect. However, donor-derived T cells also recognize non-hematopoietic cells and can damage recipient tissues such as the intestine, lungs, liver, and skin, resulting in acute graft-versus-host disease (aGvHD), which is the leading cause of non-relapse mortality and morbidity.^1^^,^^2^

We have previously reported that T cells require *nuclear factor of activated T cells* (NFAT)c1 and NFATc2 for the induction of GvHD in a mouse model of acute GvHD, whereas the GvL effect was fully preserved in the absence of either.^3^ NFATs are a family of transcription factors directly activated upon T cell receptor (TCR) engagement.^4^ An increase in intracellular Ca^2+^ concentration and the subsequent activation of calcineurin (CN) leads to CN-mediated dephosphorylation of multiple serine/threonine residues in the regulatory domain of NFAT, resulting in conformational change, exposure of nuclear localization signals, and nuclear import of cytoplasmic NFAT. T cells express NFATc1, c2, and c3 (aka NFAT2, 1, and 4), with NFATc1 and NFATc2 appearing to be more important than NFATc3 in peripheral conventional T cells (Tcon). The suppressive capacity of circulating Tregs is unaffected by NFAT single or double deficiency.^3^^,^^5^ Upon allo-HCT with NFAT-deficient T cells, Tregs are at least in part responsible for the protection against severe GvHD, while the GvL effect is preserved.^6^^,^^7^ Thus, a specific reduction of NFAT activation or gene-editing of individual NFAT members holds great promise for managing GvHD.^8^ Unlike the common strategies of CN inhibition by CsA or tacrolimus,^9^ this approach would preserve the GvL effect.

It should be noted that donor T cells are not only essential for the GvL effect but are also required to control infectious agents after allo-HCT, as infections have emerged as an important and consequential contributor to both mortality and morbidity after allo-HCT.^10^ Cytomegalovirus (CMV) infection is particularly problematic and is often implicated as a major cause of graft failure.^11^^,^^12^ Human CMV (HCMV) can destroy the bone marrow (BM) niche for hematopoiesis or infect hematopoietic stem cells (HSCs).^13^ Furthermore, HCMV infection is a major risk factor for the development of GvHD.^14^ Globally, it is estimated that 40%–80% of the population is infected with HCMV during childhood.^15^ Although HCMV infection in healthy individuals is usually subclinical, life-threatening HCMV disease is common in immunocompromised patients, and besides HCMV-mediated graft rejection and transplant failure, allo-HCT recipients may develop life-threatening pneumonia, gastroenteritis, and retinitis.^16^

CMV latency is a dormant state in immunocompetent individuals in which the virus maintains minimal gene expression but can reactivate its lytic replication and cause disease. T cells are chiefly important for immunity against HCMV because this virus drives an extraordinarily strong and persistent T cell response that dominates the memory compartment of seropositive individuals.^17^ In mice, there is a similar response to murine CMV (MCMV), which has been termed memory inflation (MI).^17^^,^^18^^,^^19^ The lifelong functionality of the immune system in CMV latency is distinctive and in juxtaposition with T cell exhaustion, the terminal differentiation into dysfunctional T cells (T_EX_) upon antigen persistence.^20^^,^^21^ Although HCMV-specific CD8^+^ T cells are also terminally differentiated, they are characterized as effector memory cells that have regained CD45RA (T_EMRA_) but downregulate the costimulatory molecules CD27 and CD28, the homing receptor CD62L, and the interleukin-7 (IL-7) receptor CD127.^22^^,^^23^ T_EMRA_ express the terminal effector-typical markers KLRG1 and CD57 as well as the chemokine receptors CXCR3 and CX3CR1.^24^ HCMV-specific CD4^+^ T cells exhibit a comparable phenotype.^25^ In contrast to CD8^+^ T_EX_, PD-1 and most other co-inhibitory receptors are not upregulated in T_EMRA_ cells. HCMV-specific CD8^+^ T cells maintain high levels of cytotoxic molecules and rapidly secrete vast amounts of IFN-γ upon activation. The persistence of functional effector cells suggests a differentiation pathway that is distinct from the formation of short-lived CD8^+^ effector T cells (SLECs) during the acute phase and from the development of T_EX_. Accordingly, a typical pattern of transcription factors characterizes MI T cells vs*.* SLEC and T_EX_.^25^

We had addressed the role of NFAT in conventional and inflated CD8^+^ T cells during acute and chronic MCMV infection. Although conventional NFAT-deficient T cells can control acute infections, *Nfatc1*^−/−^ CD8^+^ T cells were impaired and *Nfatc1*^−/−^.*Nfatc2*^−/−^ (DKO) CD8^+^ T cells were no longer able to differentiate into migrating MI cells.^26^

In the clinical transplant setting, it is possible that the recipient (R) and/or the donor (D) are latently infected with HCMV (R+). Accordingly, in 60%–70% of cases, CMV infection results from viral reactivation in CMV-seropositive allo-HCT recipients later in the posttransplant course due to profound immunosuppression.^27^ If the donor is HCMV+, HCMV-specific memory T cells are co-transferred during allo-HCT and should at least partially be available to control the reactivated HCMV. Nevertheless, 20%–30% of CMV infections after allo-HCT are caused by viral transmission from CMV-seropositive donors (D+) to CMV-seronegative recipients (R−). The most critical consideration, however, is transplanting grafts from CMV-negative donors into CMV-seropositive recipients (D− → R+), leaving the latter without appropriate T cell protection in the critical post-transplant phase. Therefore, the ability of donor T cells to control CMV infection during allo-HCT is of paramount importance.

Consistent with a fully preserved GvL effect of NFAT single-ablated T cells, model-antigen recall responses of *Nfat*^−/−^ T cells are maintained in GvHD mice.^3^ Similarly, MCMV control was largely unaffected when NFAT single-deficient CD8^+^ T cells are adoptively transferred into immunodeficient *Rag2*^−/−^*gc*^−/−^ mice.^26^ This encouraged us to further investigate whether NFAT-deficient T cells are capable of controlling acute and latent MCMV infection after allo-HCT while protecting against severe GvHD. Indeed, although acute MCMV infection of the allotransplanted mice exacerbated the degree of weight loss and clinical scores across all settings, NFAT-deficient T cells still ameliorated GvHD scores compared to wild-type (WT) T cells but could control the virus. When recipient mice were latently infected with MCMV prior to allo-HCT, weight loss and clinical GvHD scores remained lower after transplantation of NFATc1- or NFATc2-ablated T cells than with WT T cells. Different from MCMV infection in NFAT-deficient animals,^26^ upon transfer of allogeneic NFAT-ablated, especially *Nfatc1*^−/−^, T cells, we even observed a significant relative expansion of MCMV-specific KLRG1^+^CD27^–^ MI cells expressing IFN-γ and GzmB among the CD3^+^CD8^+^CD44^+^ effector T cells. We conclude that MCMV infection does not interfere with the protective effect of NFAT inhibition on GvHD development and that NFAT-deficient T cells ensure a long-term CMV control after allo-HCT.

## Results

To evaluate the impact of MCMV infection on the course of GvHD and whether T-cell-specific NFAT inhibition is still protective, we chose a haploidentical HCT setting (B6, H-2^b^ → CB6F1, H-2^b+d^). This ensures antigen presentation via syngeneic major histocompatibility complex (MHC), while providing a major mismatch for GvHD induction. In addition, haploidentical allo-HCTs are increasingly used worldwide.^28^

### Acute MCMV infection threatens mice after allo-HCT, whereas NFAT deficiency in co-transplanted T cells still partially protects against aGvHD

**Figure 1:**
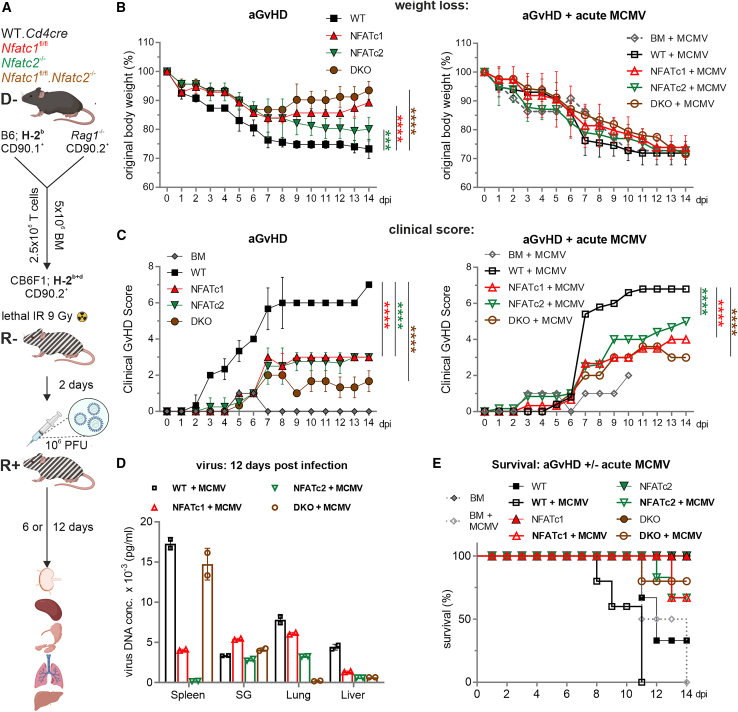
Co-transfer of single-deficient NFATc1 and NFATc2 or double-deficient NFATc1c2 T cells in a haploidentical major mismatch model limits signs of aGvHD even after an acute MCMV infection (A) Experimental *in vivo* set up of the induction of aGvHD in a haploidentical mouse model due to an H-2^b^ → H-2^b+d^ transfer with WT or NFAT-deficient T cells along with BM, preceded by lethal irradiation (9 Gy) of CB6F1 recipients. Two days post-transplantation, mice were acutely infected with 1 × 10^6^ PFU of WT-MCMV Smith strain. Six or twelve days post-infection, *ex vivo* analyses were conducted on spleen, peripheral lymph nodes (pLNs), mesenteric lymph nodes (mLNs), salivary gland (SG), lungs, and liver. (B) Weight changes were determined over 14 days post-irradiation (dpi) in mice without (aGvHD) or with an acute MCMV infection (aGvHD + acute MCMV). Mice were evaluated every day and weight loss was calculated considering day 0 weight as 100%; *n* ≥ 5. (C) Clinical scores were determined over 14 days. (B and C) Two-way ANOVA and Tukey’s multiple comparisons test (∗∗∗*p* < 0.001, ∗∗∗∗*p* < 0.0001), mean ± SEM, *n* ≥ 5, two independent experiments. (D) Virus DNA concentration of acutely infected mice 14 dpi in indicated organs was determined by RT-PCR. (E) Survival over 14 days.

In the first approach, mice were acutely infected with MCMV 2 days after allo-HCT (Figure 1A). Acute MCMV infection of the allotransplanted mice aggravated the degree of weight loss and clinical score. However, GvHD scores were less pronounced after transplantation of NFAT-deficient vs*.* WT T cells (Figures 1B, 1C, S1A, and S1B). Consistent with our earlier results in the prevention of severe aGvHD,^3^^,^^8^ the ablation of either NFATc1 or NFATc2 or the creation of a double deficiency in transplanted T cells was protective. However, over time, acute MCMV infection after allo-HCT weakened all mice, as evidenced by the steady weight loss, regardless of the presence of WT or NFAT-deficient T cells. Nevertheless, the differences in clinical GvHD scores remained genotype-specific, i.e., *Nfatc1*^−/−^ and *Nfatc2*^−/−^ T cells partially protected against aGvHD, whereas *Nfatc1*^−/−^.*Nfatc2*^−/−^ (DKO) T cells lost their distinct additional benefit compared to single deficiencies in the presence of an acute MCMV infection (Figure 1C).

Importantly, NFAT-deficient T cells were able to control MCMV at least as well as WT T cells, as measured by viral copy numbers at 6 and 12 days after acute infection (Figures 1D and S1C). Partial virus control was achieved by *Nfatc1*^−/−^ and *Nfatc2*^−/−^ T cells not only in the spleen, where transplanted T cells first appear,^29^ but also in the salivary gland (SG), a site of CMV replication, persistence, and prolonged shedding,^30^ as well as in the lungs and liver, which are organs affected by CMV.^31^^,^^32^^,^^33^

The steady weight loss upon MCMV infection was reflected in the survival rate. In the absence of MCMV, only mice receiving WT T cells started to die, which was advanced after infection. When acutely infected with MCMV, survival of mice receiving NFAT-deficient T cells was better than that of those receiving WT T cells but still not absolute (Figure 1E). In summary, although acute MCMV infection had a clear negative effect on mice receiving allo-HCT, transplantation of NFAT-deficient T cells instead of WT T cells limited the clinical severity of GvHD while still allowing viral control.

### Latent MCMV infection of allo-HCT recipients is controlled by NFAT-deficient co-transplanted T cells, which cause only mild aGvHD

**Figure 2:**
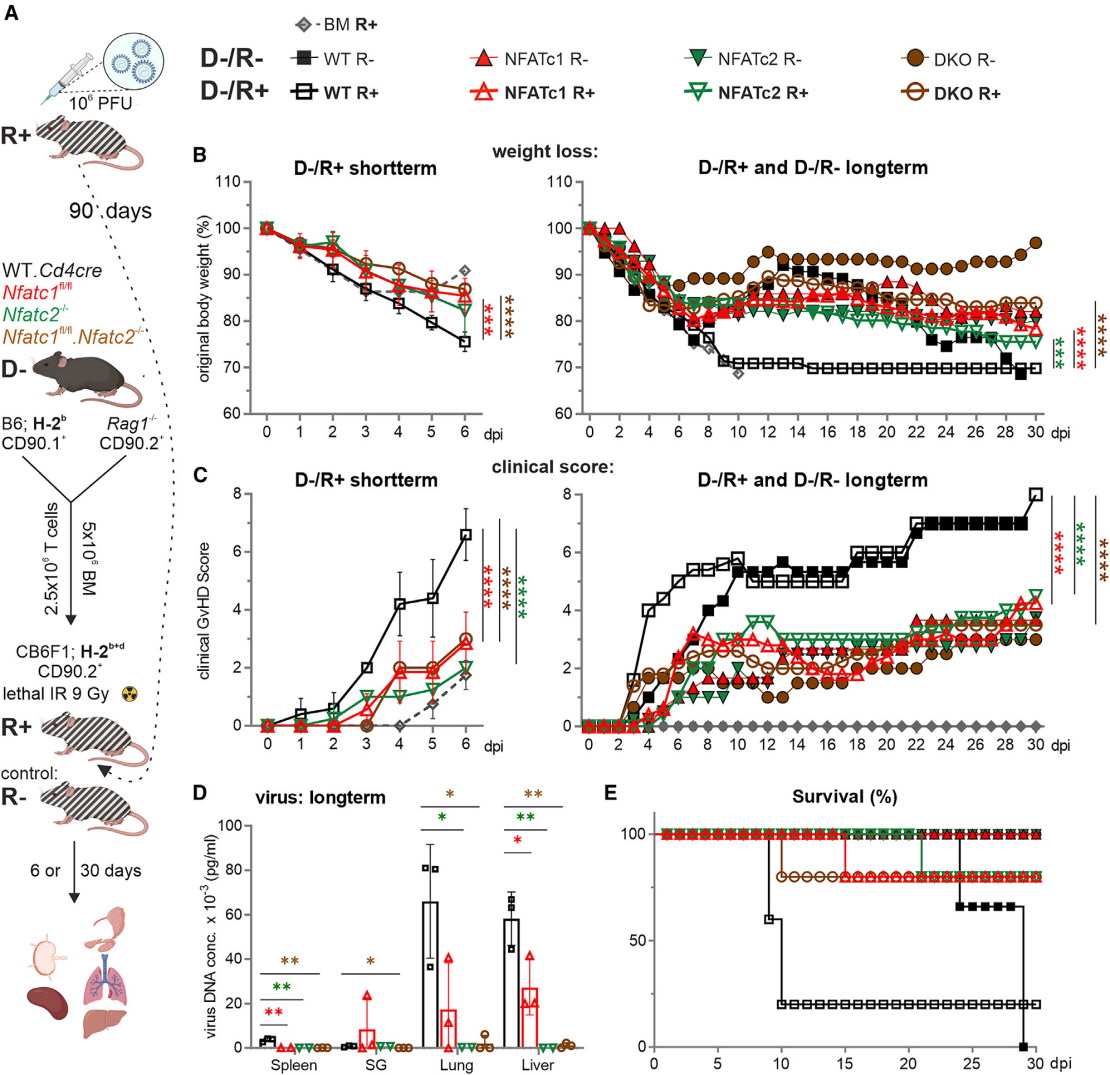
NFAT-deficient T cells can still provide protection when transferred into latently infected recipients (A) Experimental *in vivo* set up. Latent infected CB6F1 recipient (90 days prior transplantation) with WT-MCMV Smith strain. Acute GvHD was initiated by transferring H-2^b^ donor T cells together with BM cells into H-2^b+d^ recipients, preceded by lethal irradiation (9 Gy) of CB6F1 recipients. Six or thirty days post-transplantation, *ex vivo* analyses were conducted on various tissues. Symbols representing studied genotypes in only aGVHD (D−/R−) and latently infected CB6F1 recipients (D−/R+). (B) Weight loss and (C) clinical scores of GvHD-induced mice in latently infected recipients were monitored daily for short (6 dpi) or long term (30 dpi). Two-way ANOVA and Tukey’s multiple comparisons test (∗∗∗*p* < 0.001, ∗∗∗∗*p* < 0.0001), mean ± SEM, *n* ≥ 5, two independent experiments. (D) Virus DNA concentration 30 dpi, determined by RT-PCR, mean ± SD, *n* = 3, unpaired Student’s t test (∗*p* < 0.05, ∗∗*p* < 0.005). (E) Survival was monitored daily for 30 days.

To mimic a clinical high-risk scenario (D−/R+), recipients, but not donor mice, were latently infected with MCMV prior to allo-HCT (Figure 2A). Thus, allo-HCT co-transplanted T cells did not contain any MCMV-specific memory T cells. We transplanted WT, *Nfatc1*^−/−^, *Nfatc2*^−/−^, and *Nfatc1*^−/−^*Nfatc2*^−/−^ DKO T cells into unchallenged and MCMV+ mice in parallel. Reassuringly, weight loss and clinical GvHD scores remained less after transplantation of NFATc1- and/or NFATc2-ablated T cells than with WT T cells (Figures 2B, 2C, S2A, and S2B). Viral DNA increased in lungs and liver between 6 and 30 days after allo-HCT in the presence of WT T cells, whereas better control was achieved with NFAT-deficient T cells (Figures 2D and S2C). As with acute MCMV infection, the presence of MCMV decreased the survival rate after allo-HCT, most severely after transfer of WT T cells, but also occurred in groups that had received NFAT-deficient T cells (Figure 2E). In sum, in latent MCMV infection, NFAT-deficient T cells demonstrated the ability to maintain their low GvHD-inducing potential while partially controlling MCMV replication.

### NFAT-deficient Tregs enrich in MCMV+ mice during GvHD

**Figure 3:**
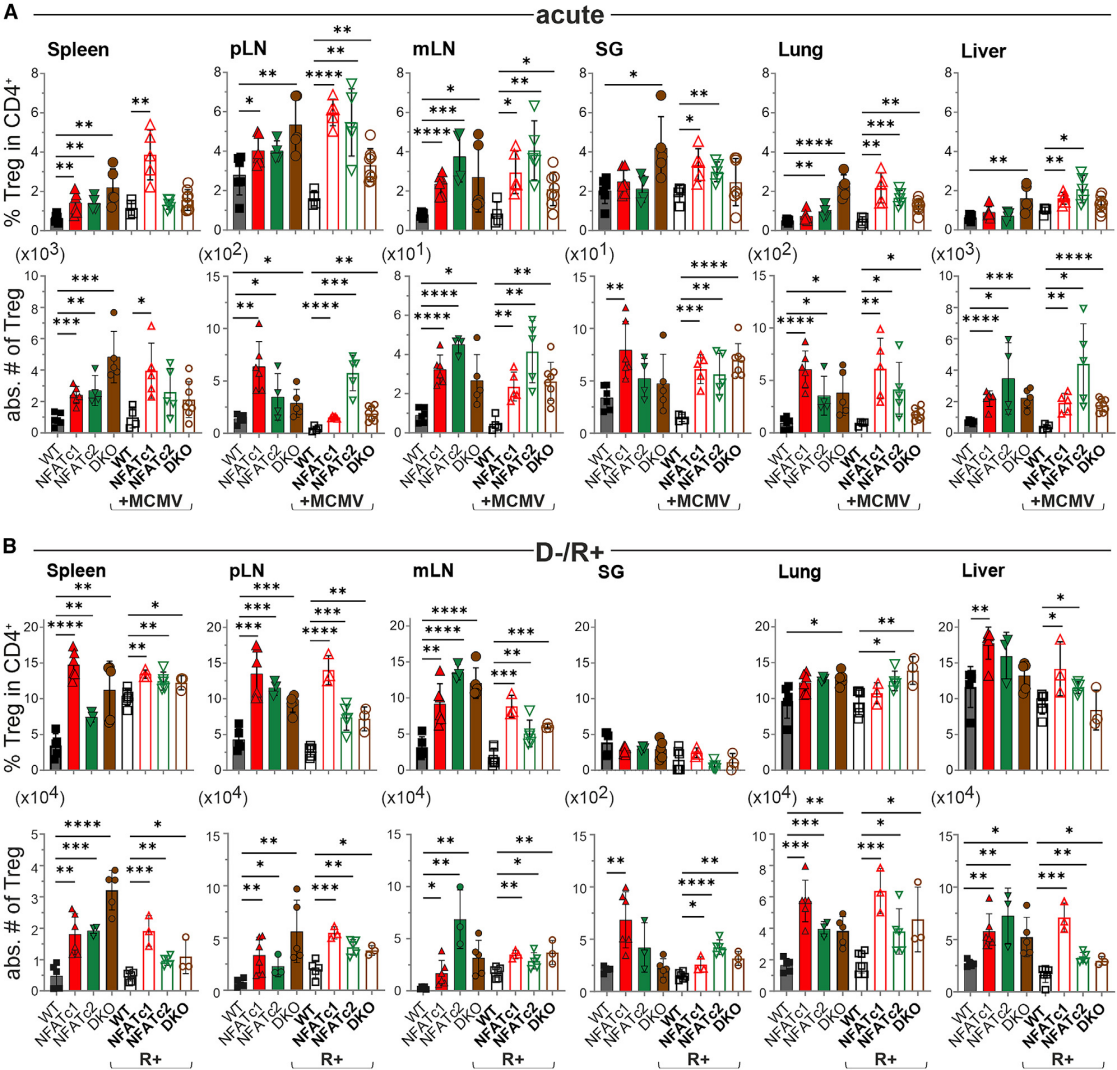
Independent from MCMV infection, NFAT-deficient Tregs are significantly enriched during GvHD (A) Frequency and absolute cell numbers—calculated back to all cells of the particular organ—of donor CD90.1^+^CD4^+^CD25^+^Foxp3^+^ T cells 8 days post-transplantation in non-infected or acutely infected (MCMV+) recipients (spleen, pLN, mLN, salivary gland [SG], lungs, and liver). Student’s two-tailed t test (∗*p* < 0.05, ∗∗*p* < 0.005, ∗∗∗*p* < 0.001, ∗∗∗∗*p* < 0.0001), mean ± SEM, *n* ≥ 4, three independent experiments. (B) Flow cytometric analyses of indicated organs harvested 6 days post-transplantation from mice without or with latent MCMV infection (R+). Percentage of CD25^+^Foxp3^+^ Tregs within donor CD90.1^+^CD4^+^ T cells and absolute cell numbers. (A and B) Student’s two-tailed t test (∗*p* < 0.05, ∗∗*p* < 0.005, ∗∗∗*p* < 0.001, ∗∗∗∗*p* < 0.0001), mean ± SEM, *n* ≥ 3, two independent experiments.

Further analyses were conducted using flow cytometry on T cells from various organs (Figures S3A and S3B). In the context of a complete major mismatch model without MCMV infection, we had found that transfer of NFAT-deficient T cells (conventional plus regulatory CD3^+^ T cells) allow an increase in the frequency of protective Treg cells.^3^^,^^8^ In all settings, without vs*.* acute or latent MCMV infection, the frequencies of NFAT-deficient Tregs in total CD4^+^ T cells as well as their absolute cell numbers increased also in the haploidentical GvHD model (Figures 3A and 3B).

### Irrespective of MCMV infection, NFAT-deficient CD4^+^ and CD8^+^ T cells produce fewer proinflammatory cytokines during GvHD

NFAT was first described as a transactivator of cytokine genes. Accordingly, both CD4^+^ and CD8^+^ NFAT single- or double-deficient T cells expressed less proinflammatory cytokines like IFN-γ during aGvHD,^3^^,^^8^ and this was similar when mice were acutely infected with MCMV after allo-HCT (Figures S4A and S4B).

**Figure 4:**
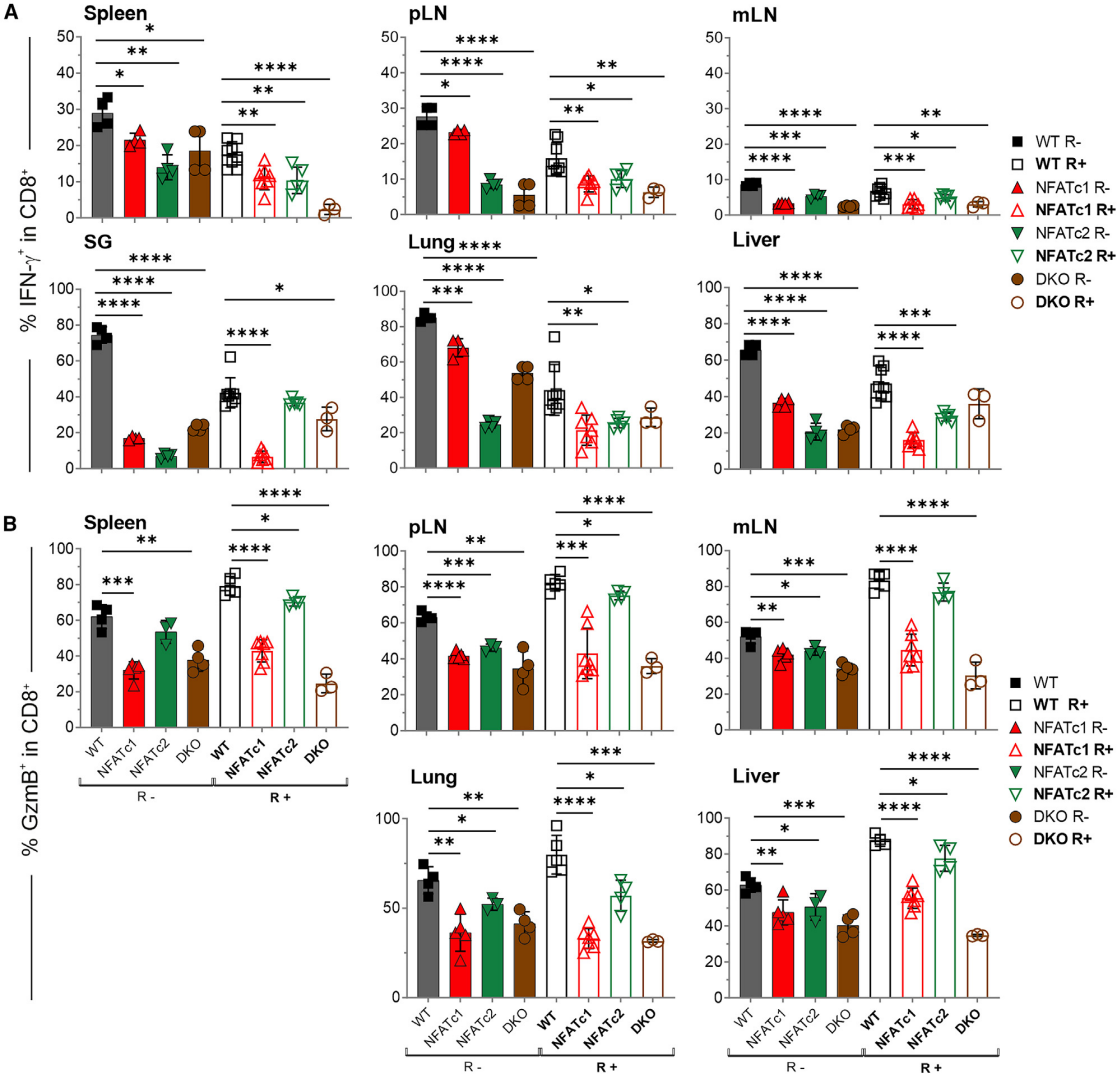
The reduction of proinflammatory cytokines and cytotoxic molecules in NFAT-deficient CD8^+^ T cells during aGvHD remains significant regardless of a concurrent MCMV infection (A and B) Flow cytometric analyses of recipient mice without (R−) and with latent MCMV infection (R+) were conducted 6 days post-transplantation. (A) Frequency of donor CD90.1^+^CD8^+^IFN-γ^+^ T cells in the depicted organs, *n* ≥ 3. (B) Frequency of donor CD90.1^+^CD8^+^GzmB^+^ T cells. (A and B) Student’s two-tailed t test (∗*p* < 0.05, ∗∗*p* < 0.005, ∗∗∗*p* < 0.001, ∗∗∗∗*p* < 0.0001), mean ± SEM, *n* ≥ 3, two independent experiments.

As frequently observed during immune reconstitution after allo-HCT, CD8^+^ T cells dominated over CD4^+^ T cells in GvHD mice (Figure S5A). In latently infected mice, *Nfatc1*^−/−^ and *Nfatc1*^−/−^*Nfatc2*^−/−^ (DKO) CD8^+^ T cells were relatively more abundant, whereas *Nfatc2*^−/−^ CD4^+^ T cells reached an almost equal distribution within the CD90.1^+^ donor T cell population. Despite some minor effects of the individual NFAT deficiency, the vast majority of both CD4^+^ and CD8^+^ T cells exhibited an effector CD44^+^CD62L^–^ phenotype yet expressed less IFN-γ when NFAT-deficient (Figures 4A, S5B, and S5C). Tumor necrosis factor (TNF), although detectable in far fewer CD8^+^ T cells than IFN-γ, followed the same pattern (Figure S6A).

The dominance of IFN-γ expression and its reduction upon transplantation of NFAT-deficient T cells could be confirmed in sera (Figure S7A). IFN-γ production can originate not only from type 1 lymphocytes but also from pathogenic IFN-γ/IL-17 co-producers.^34^ IL-17 was barely detectable in the sera, but the ratio of IFN-γ to IL-17 changed significantly due to reduced IFN-γ secretion in latently MCMV-infected mice transplanted with *Nfatc2*^−/−^ and especially *Nfatc1*^−/−^ or DKO T cells (Figure S7B). CD4^+^ and CD8^+^ IL-17^+^ as well as IFN-γ^+^/IL-17^+^ T cells were underrepresented in lymphoid organs, whereas SG, liver, and especially the lungs harbored very small but distinct populations in GvHD-diseased mice (Figures S7C and S8A). Irrespective of an MCMV infection, NFAT-deficient T cells expressed less IL-17, although their frequency was increased in *Nfatc1*^−/−^ vs*.* WT T cells in the liver. However, the ratio of the absolute numbers of all T-helper (Th) 17 or T-cytotoxic (Tc) 17 to CD4^+^ Treg cells was still in favor of Tregs in the liver like in the lymphoid organs (Figure S8B). Apart from *Nfatc1*^−/−^ T cells in the liver of latently MCMV-infected GvHD mice, NFAT-deficient T cells promoted a shift toward Tregs.

In allotransplanted mice, granzyme B (GzmB)-positive CD8^+^ donor T cells increased in frequency in latently MCMV-infected in comparison to uninfected animals (Figure 4B). However, NFAT-deficient—especially *Nfatc1*^−/−^ and DKO CD8^+^ T cells—were unable to fully express GzmB and perforin (Prf1) (Figures 4B and S6B).

In conclusion, consistent with preserved reduced clinical GvHD scores upon transplantation of NFAT-deficient T cells in the presence of MCMV infection, the donor T cells produced less IFN-γ, TNF, GzmB, and Prf1.

### NFAT deficiency supports terminal differentiation of MCMV-specific CD8^+^ T cells in the context of allo-HCT

**Figure 5:**
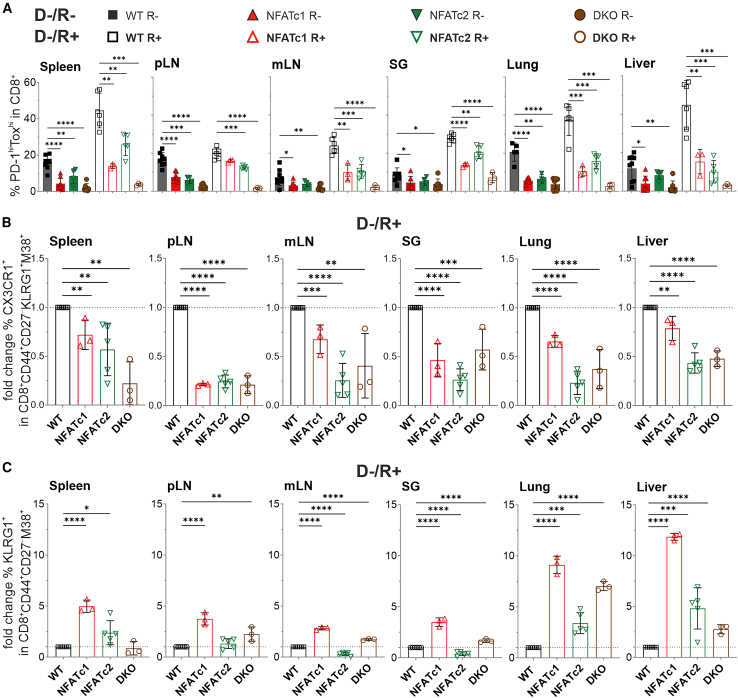
NFAT deficiency reduces CD8^+^ T cell exhaustion while promoting terminal differentiation of MCMV-specific CD8^+^ T cells after allo-HCT (A) Percentage of PD-1^hi^TOX^hi^ in CD8^+^ T cells in only aGVHD and latently infected CB6F1 recipients (+MCMV) 6 days post-transplantation, *n* ≥ 3. (B) Percentages of the chemokine receptor CX3CR1^+^ and (C) type II transmembrane protein receptor KLRG1^+^ within MCMV-specific donor CD90.1^+^CD8^+^M38^+^ T cells was determined through tetramer staining and subsequent flow cytometry analysis. Fold change was calculated relative to WT. (A, B and C) Student’s two-tailed t test (∗*p* < 0.05, ∗∗*p* < 0.005, ∗∗∗*p* < 0.001, ∗∗∗∗*p* < 0.0001), mean ± SEM, *n* ≥ 3, two independent experiments.

NFAT, especially the short isoform of NFATc1, NFATc1/αA,^35^ is heavily expressed upon chronic LCMV infection and generally believed to be part of the exhaustion program.^36^^,^^37^ To evaluate differentiation toward dysfunctional T_EX_,^38^ we stained CD8^+^ T cells with anti-PD-1, -Tox, and -Tim3 antibodies (Figure S9A). The inhibitory receptor Tim-3 marks CD8^+^ T_EX_ cells, and the transcriptional regulator Tox is essential for their epigenetic remodeling and survival although this is not exclusive for the exhausted phenotype.^37^^,^^39^ Although the percentage of PD-1^hi^Tox^hi^ CD8^+^ T cells increased in latently MCMV-infected mice receiving allo-HCT, there was no change in the number of terminally exhausted PD-1^hi^Tim3^hi^ CD8^+^ T cells in any of the organs tested (Figures 5A and S10). Importantly, *Nfatc1*^−/−^ and *Nfatc1*^−/−^*Nfatc2*^−/−^ and to a lesser extent *Nfatc2*^−/−^ could not exhaust as much as WT CD8^+^ T cells, but this was also occurring in sole GvHD animals and thus independent of MCMV infection.

Previously, we reported that MCMV-specific MI responses are particularly dependent on the expression of NFAT, especially NFATc1.^26^ To test this in the context of allo-HCT, we applied a tetramer that detects inflationary M38-specific CD8^+^ T cells.^40^ Besides by their recognized (e.g., M38) epitope, MIs are defined as CD3^+^ (here replaced by CD90.1^+^) CD8^+^CD62L^–^CD44^+^CD27^–^KLRG1^+^CX3CR1^+^ (Figure S9B). In line with our data on MCMV infection of NFAT-deficient animals,^26^ we observed a significant relative loss of CX3CR1^+^ M38-specific MI cells in all tested NFAT-deficient T cells analyzed in secondary lymphoid and non-lymphoid organs (Figure 5B). This contrasted with the proficient virus control by NFAT-deficient T cells in allotransplanted latently MCMV-infected mice. Indeed, excluding CX3CR1 from the staining protocol, we observed a highly significant fold increase of *Nfatc1*^−/−^ over WT CD44^+^CD27^–^KLRG1^+^ CD8^+^ T cells among the M38^+^ MI population (Figure 5C). Although the putative homing receptor CX3CR1 was inefficiently expressed in the absence of one or two NFAT family members, the increase in CD44^+^CD27^–^KLRG1^+^ M38^+^ MI cells was most prominent in lung and liver. Here, also *Nfatc2*^−/−^ and DKO were more prevalent than WT MIs.

Taken together, NFAT ablation resulted in less T_EX_ and MCMV-specific CX3CR1^+^ M38^+^ MI cells. Conversely, MCMV-specific CD44^+^CD27^–^KLRG1^+^ M38^+^ MI cells significantly benefited from the loss of NFAT.

### GvHD and viral control also improved with NFAT ablation in CD8^+^ T cells only

Next, we investigated whether these effects could be reproduced when NFAT deficiency was restricted to the CD8^+^ T cell pool. We allotransplanted WT CD4^+^ T cells together with either CD8^+^ WT, *Nfatc1*^−/−^, *Nfatc2*^−/−^, or DKO T cells in a 1:1 ratio into latently MCMV-infected mice (Figure S11A). Remarkably, ablation of NFAT in CD8^+^ T cells alone reduced weight loss and clinical score even though the mice generally experienced more severe symptoms in the presence of WT CD4^+^ T cells (Figures S11B and S11C). Virus control was again more efficient with NFAT-deficient T cells, here with NFAT-deficient CD8^+^ T cells (Figure S11D).

Although the number of WT CD4^+^ T cells was less affected, NFAT-deficient CD8^+^ T cells were significantly reduced in comparison to the co-transplanted WT CD8^+^ T cells (Figure S12A). In the setting with *Nfatc1*^−/−^ and DKO CD8^+^ T cells, this led to a significant shift to relatively more CD4^+^ T cells (Figure S12B), and mice with *Nfatc1*^−/−^ CD8^+^ T cells but WT CD4^+^ T cells died earliest (Figure S12C).

Consistent with the previous data, IFN-γ-, TNF-, and GzmB-positive CD8^+^ T cells were less frequent when NFATc1, NFATc2, or both were ablated (Figures S13A–S13C). In summary, although deletion of NFAT in all CD3^+^/CD90.1^+^ T cells may be superior in protecting allo-HCT recipients, NFAT ablation in CD8^+^ T cells only reduced the risk for severe GvHD while allowing MCMV control.

### NFAT-deficient MI cells express more IFN-γ and GzmB than WT MI cells

**Figure 6:**
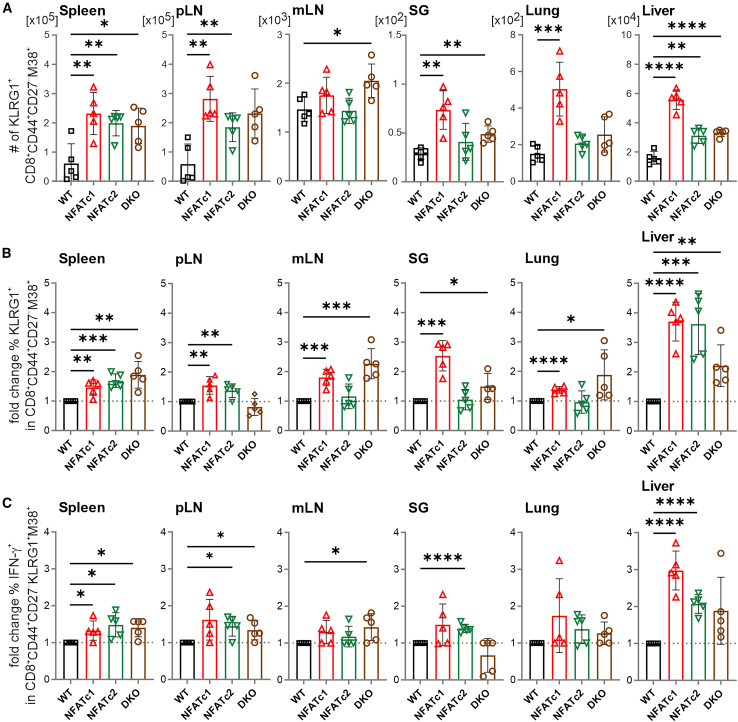
NFAT deficiency in CD8^+^ T cells is sufficient to increase the frequency of IFN-γ^+^ MI T cells (A–C) Flow cytometric analyses of allotransplanted WT CD4^+^ T cells and WT or NFAT-deficient CD8^+^ T cells (1:1 ratio) 6 days post-transplantation. (A) Absolute numbers and (B) fold change of the percentage of KLRG1^+^ within MCMV-specific donor CD90.1^+^CD8^+^CD44^+^CD27^–^M38^+^ T cells was determined through tetramer staining and subsequent flow cytometry analysis. Fold change was calculated specifically on WT. (C) Frequency of IFN-γ^+^ within MCMV-specific donor CD90.1^+^CD8^+^CD44^+^CD27^–^M38^+^ T cells (*in vitro* restimulated). (A, B and C) Student’s two-tailed t test (∗*p* < 0.05, ∗∗*p* < 0.005, ∗∗∗*p* < 0.001, ∗∗∗∗*p* < 0.0001), mean ± SEM, *n* = 5.

In line with the data from NFAT-deficient CD3^+^ T cells, NFAT-deficient CD8^+^ T cells in the presence of WT CD4^+^ T cells showed a reduced exhaustion phenotype in latently MCMV-infected animals receiving allo-HCT and developing GvHD (Figures S14A and S14B). Furthermore, in comparison to the MI epitope M38-recognizing CD3^+^/CD90.1^+^CD8^+^CD62L^–^CD44^+^CD27^–^KLRG1^+^CX3CR1^+^ WT T cells, such NFAT-deficient MI cells appeared at a significantly reduced frequency (Figure S14C). However, ignoring CX3CR1 expression again revealed an enhanced absolute number of NFAT-deficient vs*.* NFAT-sufficient CD3^+^/CD90.1^+^CD8^+^CD62L^–^CD44^+^CD27^–^KLRG1^+^ M38^+^ MI cells in secondary lymphoid and non-lymphoid organs (Figure 6A). The increase of *Nfatc1*^−/−^ and *Nfatc2*^−/−^ over WT CD27^–^KLRG1^+^ M38^+^ MI cells was approximately 3-fold in the liver (Figure 6B). In sharp contrast to the entire pool of NFAT-deficient CD8^+^ T cells, significantly more NFAT-deficient than WT M38^+^ MI cells expressed IFN-γ, which was again most pronounced in the liver (Figure 6C).

**Figure 7:**
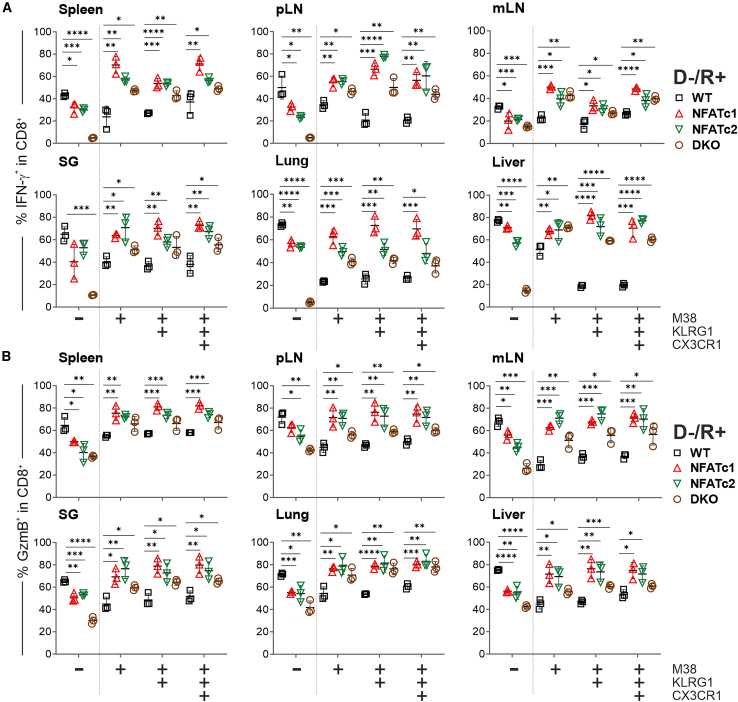
After allo-HCT in latently MCMV-infected mice, NFAT-deficient MI T cells present with a higher frequency of IFN-γ^+^ and GzmB^+^ cells Percentage of (A) IFN-γ^+^ and (B) GzmB^+^ cells of either M38^–^ or MCMV-specific M38^+^, M38^+^KLRG1^+^, and M38^+^KLRG1^+^CX3CR1^+^ CD8^+^ T cells after 6 days post-transplantation and *in vitro* restimulation. (A and B) Student’s two-tailed t test (∗*p* < 0.05, ∗∗*p* < 0.0051, ∗∗∗*p* < 0.001, ∗∗∗∗*p* < 0.0001), mean ± SEM, *n* = 3.

To verify this unexpected notion, we once more performed GvHD experiments with WT vs*.* NFAT-deficient CD3^+^ T cells and either acutely infected 2 days after allo-HCT or transplanted into latently MCMV-infected mice (Figures 1A and 2A). We evaluated IFN-γ and GzmB in M38^–^ vs*.* M38^+^ MI cells, the latter in the entire M38^+^ population, in M38^+^KLRG1^+^, and in the reduced, but still present M38^+^KLRG1^+^CX3CR1^+^ MI subpopulations (Figure S15). Of note, NFAT-deficient CX3CR1^+^ MI T cells exposed a diminished expression level of that chemokine receptor (Figure S16A). Consistent with the former results in total CD8^+^ T cells (Figure 4), NFAT-deficient conventional M38^–^IFN-γ^+^ or M38^–^GzmB^+^ CD8^+^ T cells were less frequent than their WT counterparts (Figures 7A and 7B, S17A, and S17B). However, all variants of M38^+^CD8^+^ T cells enriched in number of IFN-γ^+^ and GzmB^+^ cells as well as in the expression level of these effector molecules per cell, which was observed under both acute and latent MCMV infection during allo-HCT (Figures 7A, 7B, S16B, S16C, S17A, and S17B). *Nfatc1*^−/−^ was mostly superior to *Nfatc2*^−/−^, whereas *Nfatc1*^−/−^*Nfatc2*^−/−^ (DKO) was least effective, but still resulted in more IFN-γ^+^ and GzmB^+^ M38^+^CD8^+^ MI T cells than the transplantation with WT T cells.

Therefore, in general, T cells lacking NFATc1 and/or NFATc2 exhibited a reduced proinflammatory and cytotoxic phenotype during allo-HCT and MCMV infection. In contrast, the MCMV-specific M38^+^CD8^+^ T cell subpopulation lacking NFAT not only exhibited an increase in number but also demonstrated an enhanced frequency of IFN-γ^+^ and GzmB^+^ T cells.

## Discussion

In a mouse model of allo-HCT with MHC major mismatch (H2^b^→H2^d^), NFAT single-deficient vs*.* WT T cells ameliorate GvHD but preserve the GvL effect.^3^ Here, we investigated the effect of MCMV infection in a haploidentical model (H2^b^→H2^b+d^). As expected, T-cell-specific NFAT ablation was also protective in the parent-to-F1 setting. Although MCMV infection of allotransplanted mice definitely exacerbated the clinical outcome, the transfer of NFAT-deficient T cells still improved GvHD scores compared to WT T cells.

Provoked by conditioning-induced tissue damage, tissue-derived antigens are presented by the MHC molecules and recognized as alloantigens. Supported by co-stimulation and cytokine signaling, donor T cells undergo activation, proliferation, and migrate to target organs. CD4^+^ and CD8^+^ T cells respond as type I differentiated T cells producing IFN-γ, TNF-α, and cytotoxic mediators such as Prf1 and GzmB, which drive the pathogenesis of aGvHD.^41^^,^^42^ Although polyclonally activated T cells do not necessarily reflect alloreactivity, fewer type I responsive T cells after NFAT ablation might contribute to less severe GvHD and advocate for reducing NFAT expression as a valuable option in the prevention of severe GvHD.

NFAT was discovered as a transactivator of IL-2 and subsequently recognized as essential for the expression of most cytokines.^4^^,^^43^ Therefore, reduced IFN-γ and TNF-α expression in NFAT-deficient T cells followed predictions and what we had previously described in the context of GvHD induction.^3^^,^^8^ The degree of GzmB expression after allo-HCT is T-cell-subtype-dependent.^42^ Furthermore, NFAT-mediated GzmB and Prf1 expression appears to be context-dependent, as their expression was only minimally affected in *Nfatc1*^−/−^ or *Nfatc2*^−/−^ CD8^+^ T cells when transplanted in a major mismatch model.^3^ However, in the absence of GvHD, such NFAT-ablated CD8^+^ T cells respond to an acute MCMV infection with a significant loss of *Gzma*, *Gzmb*, and *Prf1* transcripts.^26^ Consistent with this, ChIP-seq experiments revealed that regions far upstream and immediately downstream of the *Gzmb* gene, as well as the proximal promoter of the *Prf1* gene, bind NFATc1 and NFATc2 in acutely activated CD8^+^ T cells.^44^^,^^45^ The proximal promoter of *Gzmb* contains a weak non-canonical NFAT-response element,^46^^,^^47^ but binding sites for other transcription factors have been identified in the *Gzmb* promoter as well as multiple possible interaction partners for NFATc1 and NFATc2.^48^^,^^49^ Consistent with a context-dependent fine-tuning of NFAT-mediated GzmB expression, AP-1, Ikaros, and CREB emerged in both studies. Interestingly, such response elements are also found in the 1kb enhancer of *Prf1*.^50^

Another finding of our study was that MCMV could be partially controlled by MCMV-unexperienced T cells after allo-HCT, and control of viral load was better with NFAT-deficient than with WT T cells. This correlated not only with a relative increase in the number of MCMV-specific NFAT-deficient CD8^+^ MI cells but also with a heightened frequency of IFN-γ^+^ and GzmB^+^ MIs. Apparently, GzmB and IFN-γ are wired differently in inflated than in conventional CD8^+^ T cells. This is in line with the epigenetic changes observed in human T_EMRA_ and the frequent closure of promoter/enhancer regions.^51^ As terminally differentiated effector T cells, human T_EMRA_ and murine MIs express high levels of T-bet, Blimp-1, and Hobit, together enabling effector functions.^25^^,^^52^ The described ratio of T-bet^hi^ to Eomes^int^ alone excludes an exhausted phenotype, but empowers the elevated expression of IFN-γ, TNF-α, GzmB, and Prf1,^22^^,^^53^ making the contribution of NFAT to their expression negligible.

A need for sufficient NFAT levels is nevertheless expected for MIs because high-affinity TCRs drive memory inflation and recurrent TCR stimulation is required to maintain MI cells.^54^^,^^55^ Already the priming of MI T cells has been shown to be less dependent on CD28 signaling than conventional CD8 responses,^56^ whereas CMV-specific memory inflation is driven by antigen-presentation on non-hematopoietic cells in lymph nodes.^57^ Consistent with the presumed dominance of TCR signaling, but opposite to the observations here in the context of allo-HCT, NFATc1 is essential for inflationary responses during MCMV infection.^26^ In NFAT single-deficient T cells, two T-cell-expressed NFAT members were still present and in the *Nfatc1*^−/−^*Nfatc2*^−/−^ DKO at least NFATc3, which seemed to be partially sufficient when expressed in the context of low/no co-stimulation and severe inflammation caused by aGvHD.

MIs are recruited as CX3CR1^+^CD8^+^ T cells by CX3CL1, which is released from inflamed blood vessels, to sites of latent infection and expand on non-hematopoietic cells in IL-15-rich niches.^58^ As previously described,^26^ CX3CR1 is dependent on the presence of NFAT, which is in line with *Cx3cr1* being a direct NFAT target gene.^45^ Since it has been reported that memory inflation can be unaltered in the case of CX3CR1 ablation,^59^ migration of MIs may be compensated by other mechanisms as in the context of GvHD T cell homing may be facilitated by alloreactivity and inflammatory conditions, as well as a more severe MCMV infection than in healthy mice. Chemokine receptors that have been described to facilitate migration to the liver and lungs, respectively, during infection are CCR5 and CCR4.^60^^,^^61^ Notably, fold increase in KLRG1^+^ M38^+^ T cells was highest in these latter organs.

CX3CR1^int^ CD8^+^ T cells as found here are particularly relevant for MI formation as they combine the effector-memory phenotype with retained self-renewal capacity.^62^ Therefore, the intermediate expression of CX3CR1 on NFAT-deficient MI cells, associated with enhanced proliferation, explains their increase in number. In general, CX3CR1^int^ CD8^+^ T cells are the predominant T cell memory subset surveying peripheral tissues,^63^ giving them the chance to home to lungs and liver as CX3CR1^int^ MI T cells, where latent CMV is reactivated due to conditioning and aGvHD. Here, they also benefit from the IL-15-rich niches, which they might not be able to reach in the absence of such a proinflammatory state.^26^

Reducing the level of NFAT expression may be beneficial on another level. Prolonged exposure to a chronic viral infection and persistent TCR engagement fuels an inflated memory in case of repetitive TCR engagements or causes T cell exhaustion upon constant TCR signaling. During the negative feedback program of exhaustion, lack of co-stimulation and chronic TCR engagement results in robust NFAT activation and in vast amounts of nuclear NFAT alone or in atypical complexes.^64^ In line with a central role of NFAT in T_EX_ differentiation, we observed an overall reduction of markers associated with exhaustion in NFAT-deficient CD8^+^ T cells. At first glance, this may prompt concerns in the context of GvHD, but since the conventional T cells secreted less IFN-γ, TNF-α, Prf1, and GzmB and since NFAT-deficient Tregs—being functional^3^^,^^5^—were enriched, this does not. This is particularly important for NFAT single-deficient non-exhausted T cells with reduced, but certainly not complete, loss of function and the associated more pronounced increase in *Nfatc1*^−/−^ or *Nfatc2*^−/−^ Tregs than in their DKO counterparts, i.e. NFAT-deficient Tregs control the less exhausted NFAT-deficient Tcon. That donor Tregs *per se* are beneficial for GvHD also in latently MCMV-infected mice, we had demonstrated before, as an adoptive transfer of WT Tregs resulted in a significant reduction in GvHD severity, facilitated lymphoid reconstitution, while effectively controlling MCMV reactivation and dissemination, and ultimately improved survival outcomes of transplanted recipients.^65^ As a possibility, lowering the level of NFAT may even strengthen the MI phenotype on the expense of T-cell differentiation toward exhaustion, driven by an overshooting reactivating viral infection, allowing a lasting control of MCMV during GvHD.

CNIs like CsA and tacrolimus are routinely administered to prevent and treat GvHD during allo-HCT. Unfortunately, CNI therapy completely inhibits T cells including Tregs and thus carries the risk of CMV reactivation. It seems that knocking out one NFAT family member in transplanted T cells would still ameliorate GvHD but allow control of reactivated or acutely transmitted CMV. One option is to gene-edit the T cells with CRISPR/Cas9 prior to infusion.^8^ The next step to study should be the interplay between antiviral letermovir prophylaxis^66^^,^^67^ and ablation of individual NFATs in the prevention of severe GvHD and control of CMV.

### Limitations of the study

This particular model of MCMV and GvHD does not encompass all the relevant clinical aspects. Mice maintained under specific-pathogen-free (SPF) conditions may present with an immunological naive state, a characteristic that differs from that of human patients. Thus, the latent MCMV infection in the recipient mice served as the sole stimulant of the immune system. Furthermore, the optimal clinical scenario (D+/R+) would entail the utilization of T cells derived from CMV-experienced individuals, a hypothesis that remains to be examined in this study.

## Resource availability

### Lead contact

Further information and requests for resources and reagents should be directed to, and will be fulfilled by, the lead contact Friederike Berberich-Siebelt (path230@mail.uni-wuerzburg.de).

### Materials availability

This study did not generate new unique reagents.

Mice bred and used in this study will be made available on request, but we may require a completed materials transfer agreement with us and others.

### Data and code availability

• All data presented in this manuscript are accessible in the Supporting Data Values XLS file.
• Any additional information required to reanalyze the data reported in this article is available from the lead contact upon reasonable request.
• This study did not generate new data or code.

## Acknowledgments

We thank Benjamin Lunz for excellent experimental support and Stefan Klein-Hessling for sharing his expertise on RT-qPCRs. We also thank Manivel Lodha for providing MCMV stocks and Andreas Beilhack’s research group, especially Juan Gamboa Vargas, for providing male B6aJ mice for breeding.

This work was supported by the 10.13039/501100001659Deutsche Forschungsgemeinschaft (DFG, German Research Foundation), DFG/FOR 2830 (L.C.-S and F.B.-S.). Additional funding was received from the DFG project number 324392634–TRR 221, B0 (F.B.-S.) and Z01 (A.R.) and DFG/BE2309/8-1 (F.B.-S.) and the German Cancer Aid/70114946 (F.B.-S.).

## Author contributions

N.H. designed and conducted experiments and acquired and analyzed data; S.M., Y.X., S.S.H., H.G., C.M.C., and R.S. performed research; A.R. provided resources; L.C.-S. and F.B.-S. conceived the project; F.B.-S. designed and organized the research; N.H., M.E., L.C.-S., and F.B.-S. interpreted data. F.B.-S. drafted the manuscript with N.H.'s participation, while S.M. and M.E. participated in extended proof-readings.

## Declaration of interests

The authors declare no competing interests.

## Declaration of generative AI and AI-assisted technologies in the writing process

During the preparation of this work, the corresponding author used https://www.deepl.com/de/write in order to check grammar, spelling, and readability. After using this tool/service, all authors reviewed and edited the content as needed and take full responsibility for the content of the published article.

## STAR★Methods

### Key resources table

**Table:** 

REAGENT or RESOURCE	SOURCE	IDENTIFIER
**Antibodies**		

anti-mouse FcgRII/FcgRIII (2.4G2)	BD Biosciences	Cat#: 553141; RRID:AB_394656
anti-mouse CD4 (GK1.5)	BioLegend	Cat#: 100414; RRID:AB_312699
anti-mouse CD4 (GK1.5)	BioLegend	Cat#: 100428; RRID:AB_493647
anti-mouse CD4 (GK1.5)	BioLegend	Cat#: 100422; RRID:AB_312707
anti-mouse CD4 (RM4:5)	BioLegend	Cat#: 100538; RRID:AB_893325
anti-mouse CD8α (53-6.7)	BioLegend	Cat#: 100752; RRID:AB_2563057
anti-mouse CD8α (53-6.7)	BioLegend	Cat#: 100722; RRID:AB_312761
anti-mouse CD8α (53-6.7)	BioLegend	Cat#: 100731, RRID:AB_893427
anti-mouse CD8α (KT15)	Thermo Fisher Scientific, Invitrogen	Cat#: MA5-16759; RRID:AB_2538242
anti-mouse CD25 (PC61)	BioLegend	Cat#: 102026; RRID:AB_830745
anti-mouse CD25 (7D4)	BD	Cat# 553070; RRID:AB_394602
anti-mouse CD27 (LG.3A10)	BioLegend	Cat#: 124218; RRID:AB_2561546
anti-mouse CD27 (LG.3A10)	BioLegend	Cat#: 124214; RRID:AB_2275577
anti-mouse CD44 (IM7)	BioLegend	Cat#: 103030; RRID:AB_830787
anti-mouse CD62L (MEL-14)	BioLegend	Cat#: 104430; RRID:AB_2187124
anti-mouse CD90.1 (OX-7)	BioLegend	Cat#: 202520; RRID:AB_2303153
anti-mouse CX3CR1 (SA011F11)	BioLegend	Cat#: 149008; RRID:AB_2564492
anti-mouse KLRG1 (2F1/KLRG1)	BioLegend	Cat#: 138421; RRID:AB_2563800
anti-mouse Tim3 (B8.2)	BioLegend	Cat#: 134011; RRID:AB_2632735
anti-mouse Lag3 (C9B7W)	BioLegend	Cat#: 125210; RRID:AB_10639727
anti-mouse PD-1 (RMP1-30)	eBioscience	Cat#: 11-998181
anti-mouse Foxp3 (FJK-16s)	eBioscience	Cat#: 17-5773-82; RRID:AB_469457
anti-mouse TOX (REA473)	Miltenyi	Cat#: 130-120-785; RRID:AB_2801785
anti-mouse IFN-γ (XMG1.2)	BioLegend	Cat#: 505841; RRID:AB_2562187
anti-mouse IFN-γ (XMG1.2)	BioLegend	Cat#: 505808; RRID:AB_315402
anti-mouse IL-17A (TC11-18H10.1)	BioLegend	Cat# 506916; RRID:AB_536018
Steptavidin PE-Cy7	BioLegend	Cat#: 405206
anti-mouse TNF-α (B8.2)	BioLegend	Cat#: 506321; RRID:AB_961435
anti-mouse/human Granzyme B (GB1)	BioLegend	Cat#: 515408; RRID:AB_2562196
anti-mouse Perforin (eBio0MAK-D)	eBioscience	Cat#: 17-9392-80; RRID:AB_469514
MCMV M38_316-323_ MHC class I -peptide complexes fluorophore-conjugated (PE) tetramer	NIH tetramer core facility	IEDB_Reference:1005878

**Bacterial and virus strains**		

WT MCMV	pSM3fr-MCK-2fL clone 3.3^68^	Smith Strain

**Chemicals, peptides, and recombinant proteins**		

10 x permeabilization	Invitrogen	00-8333-56
Bovine Serum Albumin (BSA)	Sigma-Aldrich	A9418-50G
Brefeldin A (1000x)	eBioscience	00-4506-51
CaCl_2_	Roth	CN93.1
Collagenase D	Roche	11088882001
DMEM	Gibco	10500–064
DNase I	Sigma	11284932001
10x PBS pH 7.2	LiRockland	MB-008
Fetal Bovine Serum (FBS)	Gibco	10082–147
Ionomycin	Sigma	I0634
Monensin (1000x)	eBioscience	00-4505-51
DPBS	PAN Biotech	P04-36500
Power SYBR™ Green PCR Master Mix	Thermo Fisher Scientific	4367659
Percoll	Cytiva	17089101
Phorbol 12-myristate 13-acetate	Sigma-Aldrich	79346-1MG
RPMI1640	Gibco	21875–059
Ketable (Ketamine hydrochloride)	Bela-Pharm	402581.0000
Xylavet (20 mg/mL)	CP-pharma	401510.0000
Baytril	Bayer	13113.00.02

**Critical commercial assays**		

Foxp3/Transcription Factor staining kit	eBioscience	00-521-00
IC Fixation Buffer kit	eBioscience	00-8222-49
MojoSort™ mouse CD3 T cell Isolation Kit	BioLegend	480031
MojoSort™ mouse CD4 T cell Isolation Kit	BioLegend	480033
MojoSort™ mouse CD8 T cell Isolation Kit	BioLegend	480035
*Quick*-DNA Miniprep Plus Kit	Zymo Research	D4069
LEGENDplex MU Th Cytokine Panel	BioLegend	741044

**Experimental models: Organisms/strains**		

B6J.*Cd4cre*.*Cd90.1*.*luc*	In house	N/A
B6J.*Nfatc1*^fl/fl^.*Cd4cre*.*Cd90.1*.*luc*	In house	N/A
B6J.*Nfatc2*^−/−^.*Cd4cre*.*Cd90.1*.*luc*	In house	N/A
B6J.*Nfatc1*^fl/fl^.*Nfatc2*^−/−^.*Cd4cre*.*Cd90.1*.*luc*	In house	N/A
*Rag1*^−/−^	In house	N/A

**Oligonucleotides**		

Actb qPCR Forward primer	GACGGCCAGGTCATCACTATTG	N/A
Actb qPCR Reverse primer	AGGAAGGCTGGAAAAGAGCC	N/A
MCMV gB qPCR Forward Primer	^69^ F:GCAGTCTAGTCGCTTTCTGC	N/A
MCMV gB qPCR Reverse Primer	^69^R:AAGGCGTGGACTAGCGATAA	N/A
Pthrp qPCR Forward Primer	^69^ F:GGTATCTGCCCTCATCGTCTG	N/A
Pthrp qPCR Reverse Primer	^69^ R:CGTTTCTTCCTCCACCATCTG	N/A

**Software and algorithms**		

FlowJo	Tree Star	10.8.1
GraphPad Prism	Graphpad	V.5
CorelDRAW	Corel	2021

**Quantification and statistical analysis**		

GraphPad Prism	Unpaired Student’s t test (∗*p* < 0.05, ∗∗*p* < 0.005, ∗∗∗*p* < 0.001, ∗∗∗∗*p* < 0.0001)	V.5
GraphPad Prism	two-way ANOVA Tukey’s multiple comparisons (∗*p* < 0.05, ∗∗∗*p* < 0.001, ∗∗∗∗*p* < 0.0001)	V.5

### Experimental model and study participant details

#### Mice

For the generation of B6(Cg)-Tyrc-2J/J.BALB/c F1 [Albino CB6F1, CD90.2^+^ H-2^b+d^] recipient mice, B6(Cg)-Tyrc-2J/J [Albino, H-2^b^] mice, mutated in the gene for tyrosinase were crossed with BALB/c [H-2^d^] mice.^70^ Subsequently, B6J.*Cd4cre*.*Cd90.1*.*luc* transgenic mice ubiquitously expressing firefly luciferase and CD90.1 as a congenic marker^29^ were cross-bred to generate T cell donors with *Nfatc1*^fl/fl^.*Cd4cre* [B6J.*Nfatc1*^fl/fl^.*Cd4cre*.*Cd90.1*.*luc*], *Nfatc2*^−/−^ [B6J.*Nfatc2*^−/−^.*Cd4cre*.*Cd90.1*.*luc*] and *Nfatc1*^fl/fl^*Nfatc2*^−/−^.*Cd4cre* [B6J.*Nfatc1*^fl/fl^.*Nfatc2*^−/−^.*Cd4cre*.*Cd90.1*.*luc*]. BM donor *Rag1*^−/−,^^71^ were on the C57BL/6J background (H-2^b^) and used for experiments between 8 and 12 weeks of age. All mice, male or female, were bred and maintained at the ZEMM and in the Institute of Hygiene and Microbiology, University of Würzburg.

#### Study approval

Ethical approval for all animal experiments was obtained from the appropriate authorities (Gov. Lower Franconia, file number 55.2-2532-2-835) and adhered to German animal protection regulations.

For allo-HCT, approximately equal numbers of female and male animals were used as recipients. No sedentary mouse was included. To avoid bias due to injuries caused by fighting and aggressive behavior in the cages, male mice were kept in groups as weaned from their mothers. Animals for BM and T cell collection were usually male, so that both male and female mice could be transplanted at the same time; that is because T cells from male mice are tolerant to Y and X chromosome-specific expressions. However, in some experiments, only female donor and recipient mice were used. No sex bias was found in the results.

### Method details

#### BM and T cell isolation

BM cells were obtained by flushing the femur and tibia bones of Rag1^−/−^ mice with PBS containing 0.1% BSA and filtering through a 70 μm cell strainer. Spleens and lymph nodes were likewise filtered through a 70 μm cell strainer, washed with PBS containing 0.1% BSA. For the enrichment of CD3^+^ T cells there was the use of the Mojosort Mouse CD3 T cell Negative Isolation kit (Biolegend, cat. no. 480024) following the manufacturer’s instructions. CD4^+^ or CD8^+^ T cells were subsequently isolated using the negative Isolation kit (480033 or 480035, Biolegend) according to the manufacturer’s instructions.^3^

#### Allogenic hematopoietic stem cell transplantation

CB6F1 recipient mice (CD90.2^+^ H-2^b+d^) underwent myeloablative total body irradiation (TBI) at a dosage of 9.0 Gy administered with the Faxitron TT-160-CP X-ray system. Two hours after irradiation mice were retro-orbitally injected with 5 x 10^6^ BM cells from *Rag*^−/−^ mice (H-2^b^ CD90.2^+^) together with 2.5 × 10^6^ T cells from B6J.*Cd4cre*.*Cd90.1*.*luc* with distinct genotypes (B6J.*Nfatc1*^fl/fl^.*Cd4cre*.*Cd90.1*.*luc*, B6J.*Nfatc2*^−/−^.*Cd4cre*.*Cd90.1*.*luc* and B6J.*Nfatc1*^fl/fl^.*Nfatc2*^−/−^.*Cd4cre*.*Cd90.1*.*luc*). To avoid opportunistic infections, mice were given an antibiotic (Baytril, Bayer) for a period of one week. Transplanted mice underwent daily monitoring for changes in body weight and clinical aGvHD score, which was modified based on the method described by Cooke et al..^72^

#### Virus

MCMV^WT^, which refers to the pSM3fr-MCK-2fL clone 3.3,^68^ was expanded in mouse embryo fibroblasts (MEF), purified, and quantified following established protocols.^73^ CB6F1 recipient mice were intraperitoneally infected with 1 × 10^6^ plaque-forming units (pfu) either 90 days prior to T cell transfer to induce latent infection or two days post transplantation to simulate primary infection.

#### Isolation of lymphocytes from non-lymphoid tissues

The liver was flushed via the vena cava with 10 mL of ice-cold PBS. Concurrently, the hepatic portal vein was incised to facilitate blood drainage from the liver. Subsequently, the liver tissue was carefully homogenized through a 100 μm metal cell strainer into a 50 mL falcon tube. The resulting pellet was washed twice with RPMI and centrifuged at 500 x g for 10 min at 4°C. To prepare the lungs, the thoracic and abdominal cavities were exposed. The lungs were perfused by accessing the inferior vena cava. Subsequently, 10 mL of ice-cold PBS was flushed through the right ventricle of the heart until the lungs appeared colorless. Following this, the lung tissue was finely minced and transferred to a 50 mL falcon tube containing 10 mL of digestion buffer (composed of 1 mg/mL Collagenase D, 20 μg/mL DNAse I, 5 mg/mL BSA, and RPMI) for incubation on a rotating shaker at 37°C for 20 min. The resulting lung suspensions were then filtered through a 100 μm filter into a new 50 mL tube containing RPMI, followed by centrifugation at 300 x g for 5 min at room temperature. Processing of salivary glands was conducted in a similar manner to the lung tissue. To separate lymphocytes, a 40%/80% percoll gradient centrifugation was performed for 20 min at 2000 x g (4°C, without brakes). The intermediate layer, enriched with lymphocytes, was carefully collected into a new 50 mL falcon tube filled with RPMI, followed by an additional centrifugation step at 1800 rpm for 5 min at 4°C. Sedimented cell pellets were used for flow cytometry.

#### Flow cytometry staining

Before blocking the cells with anti-FcgRII/FcgRIII (2.4G2, BD Pharmingen), cells were washed once in FACS buffer (PBS containing 0.1% BSA). Surface molecule staining was performed on ice using CD4 (APC-Cy7, Pacific Blue, PE-Cy7 [GK1.5] and PerCP [RM4:5]; Biolegend), CD8α (BV510, PE-Cy7 [53–6.7]; Biolegend and FITC []; Invitrogen), CD25 (APC-Cy7 [PC61]), CD27 (Pacific Blue, PerCP [LG.3A10]), CD44 (PE-Cy7 [IM7]), CD62L (PerCP [MEL-14]), CD90.1 (APC-Cy7 [OX-7]), CX3CR1 (APC [SA011F11]), KLRG1 (Amcyan [2F1/KLRG1]), Tim3 (PerCP-Cy5.5 [B8.2]), Lag3 (APC [C9B7W]) all from Biolegend, and PD-1 (FITC [RMP1-30]; eBioscience). Intracellular Foxp3 (APC [FJK-16s]; eBioscience), and TOX (PE [REA473]; Miltenyi) staining was performed using the Foxp3 staining kit (eBioscience) according to the manufacturer’s instructions. Intracellular cytokine staining was performed after a 6 h *in vitro* restimulation with 12-*O*-tetradecanoylphorbol-13- acetate (TPA; 10 ng/mL, Sigma) and ionomycin (5 nM, Merck Biosciences) in the presence of GolgiStop and GolgiPlug (BD Pharmingen) using the IC Fixation Buffer kit (eBioscience) for the detection of cytokines and cytotoxic effector molecules. For the staining of those IFN-γ (Amcyan, PE, [XMG1.2]; Biolegend), TNF-α (PerCP-Cy5.5 [B8.2]; Biolegend), Granzyme B (Pacific Blue [GB1]; Biolegend) and Perforin (APC [eBio0MAK-D]; eBioscience) were used. MCMV M38_316-323_ MHC class I -peptide complexes fluorophore-conjugated (PE) tetramer was provided by the NIH tetramer core facility and used after the surface staining for identification of MCMV-specific CD8^+^ T cells as described.^74^

#### Serological determinations

Blood was collected from recipient mice *ex vivo*, and cytokines were measured in serum samples using the LEGENDplex Mouse Th Cytokine Panel following the manufacturer’s protocol. This bead-based multiplex assay enables the simultaneous quantification of 12 mouse cytokines, including IL-17A and IFN-γ.

#### Quantitative qRT-PCR of viral genome load in tissues

For the evaluation of virus load, DNA was directly isolated from solid tissues using *Quick-*DNA Miniprep Plus Kit (Zymo Research) according to the manufacturer’s protocol. A recombinant plasmid standard containing sequences of both *gB* and *Pthrp* genes^75^ was used as a template to establish standard curves for quantification. Real-time qRT-PCR was performed with an ABI Prism 770 light cycler detection system using the following primer pairs specific for the viral gene M55/*gB* or the mouse genome *Pthrp*: gB-fw (5′- GCAGTCTAGTCGCTTTCTGC-3′) and gB-rev (5′-AAGGCGTGGACTAGCGATAA-3′); Pthrp-fw (5′- GGTATCTGCCCTCATCGTCTG-3′) and Pthrp-rev (5′-CGTTTCTTCCTCCACCATCTG-3′).

### Quantification and statistical analysis

The figures were generated using GraphPad Prism 5 and Corel Draw software. Group comparisons were conducted utilizing the Unpaired Student’s t-test and two-way ANOVA Tukey’s multiple comparisons test using GraphPad Prism 8 software. Statistical significance was attributed to differences with *p*-values below 0.05 (∗*p* < 0.05; ∗∗*p* < 0.005; ∗∗∗*p* < 0.001). Replicates, as indicated, represent individual mice or experiments.

## Supplemental information

Document S1. Figures S1–S17

Table S1. This XLS file contains all individual data values acquired by flow cytometry and considered for the figures, thus related to Fig. 1 B-E, Fig. 2 B-D, Fig. 3 A,B, Fig. 4 A,B, Fig. 5 A-C, Fig. 6 A-C, Fig. 7 A,B; Fig. S1 A-C, Fig. S2 A,B,D, Fig. S4 A,B, Fig. S6 A,B, Fig. S7 A,B,D, Fig. S8 A,B, Fig. S10A, Fig. S11 B-D, Fig. S12 A-C, Fig. S13 A-C, Fig. S14 A-C, Fig. S16A-C, Fig. S17 A,B
