# Supplementary material for: NFAT single-deficient murine T cells reduce the risk of aGvHD while controlling cytomegalovirus infection
Source: iScience. 2025 Feb 1;28(2):111937. doi: 10.1016/j.isci.2025.111937 (PMC11872454; doi:10.1016/j.isci.2025.111937)
Supplement: Document S1. Figures S1–S17 [file mmc1.pdf]

iScience, Volume *28*

Supplemental information

NFAT single-deficient murine T cells reduce the risk of aGvHD while controlling cytomegalovirus infection

Nadine Hundhausen, Snigdha Majumder, Yin Xiao, Sigrun S. Haeusl, Helen Goehler, Rishav Seal, Cristina M. Chiarolla, Andreas Rosenwald, Matthias Eyrich, Luka Cicin-Sain, and Friederike Berberich-Siebelt

Supplementary Figures

NFAT single-deficient murine T cells reduce the risk of aGvHD while controlling cytomegalovirus infection

Nadine Hundhausen,[1] Snigdha Majumder,[1] Yin Xiao,[1] Sigrun S. Haeusl,[1] Helen Goehler,[1] Rishav Seal,[1] Cristina M. Chiarolla,[1] Andreas Rosenwald,[1,2] Matthias Eyrich,[3] Luka Cicin-Sain,[4,5] Friederike Berberich-Siebelt[1,6*]

[1] Institute of Pathology, Julius-Maximilians-University Würzburg, Würzburg, Germany

[2] Comprehensive Cancer Centre Mainfranken, Julius-Maximilians-University Würzburg, Würzburg, Germany

[3] Department of Pediatrics, University Hospital Wuerzburg, Wuerzburg, Germany

[4] Department of Viral Immunology, Helmholtz Centre for Infection Research, Braunschweig, Germany

[5] Centre for Individualized Infection Medicine, a joint venture of Helmholtz Centre for Infection Research and Medical School Hannover, Hannover, Germany

[6] Lead Contact: path230@mail.uni-wuerzburg.de

*Correspondence: path230@mail.uni-wuerzburg.de

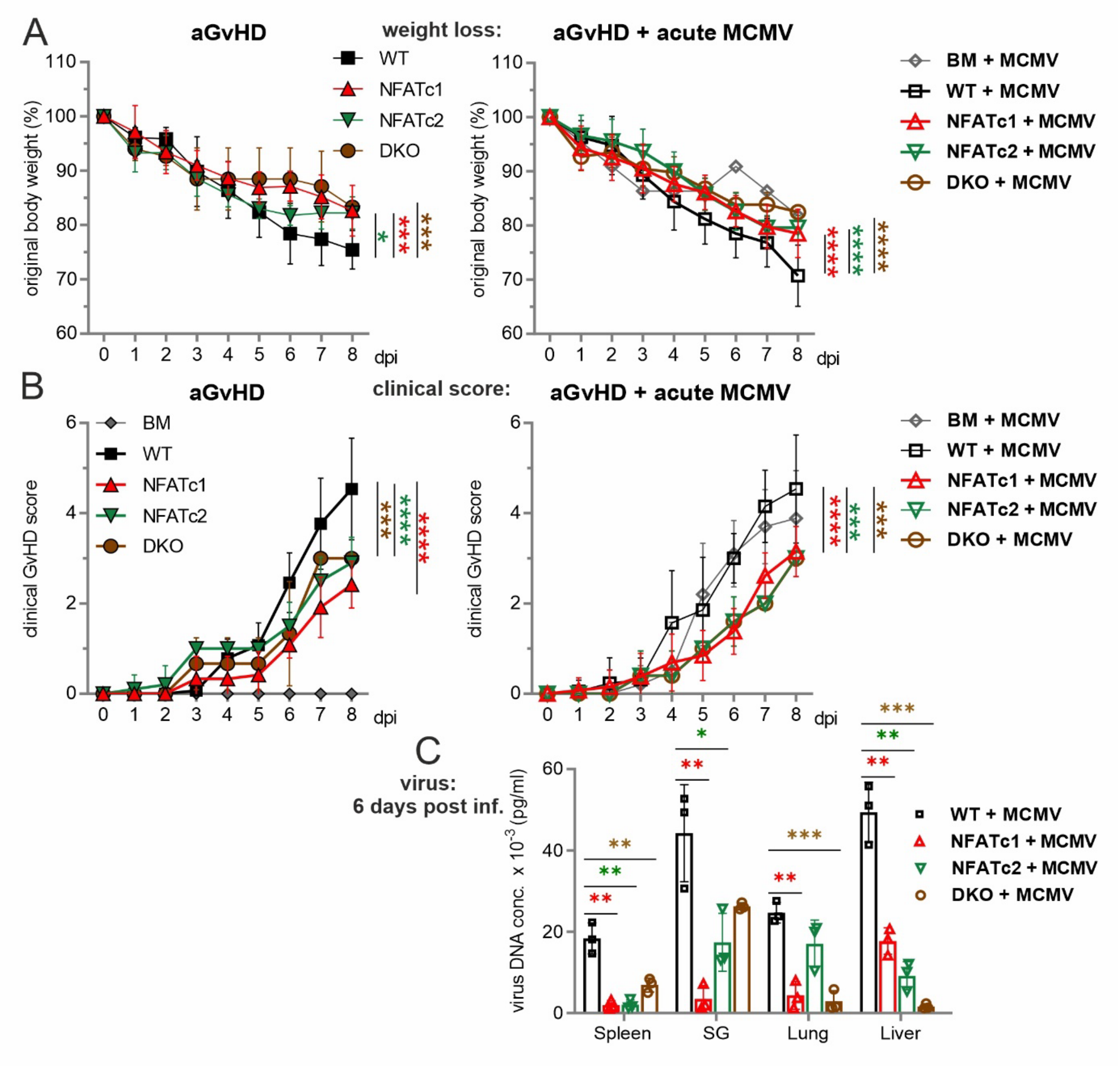

Figure S1. NFAT-deficient T cells in allo-HCT and acute MCMV infection provide protection against both aGVHD and acute MCMV infection, related to Figure 1

(**A**) Weight loss and (**B**) clinical score were determined over 8 days in mice without (aGvHD) or with an acute MCMV infection on day 2 (aGvHD + acute MCMV). Mice were evaluated every day and weight loss was calculated considering day-0 weight as 100 %. Two-way ANOVA and Tukey's multiple comparisons test (*p < 0.05, ***p < 0.001, ****p < 0.0001), mean ± SEM, n≥5, three independent experiments. (**C**) Virus DNA concentration was determined by RT-PCR 8 days post transplantation, mean ± SD, n=3, unpaired Student's t test (*p < 0.05, **p < 0.005, ***p < 0.001), two independent experiments.

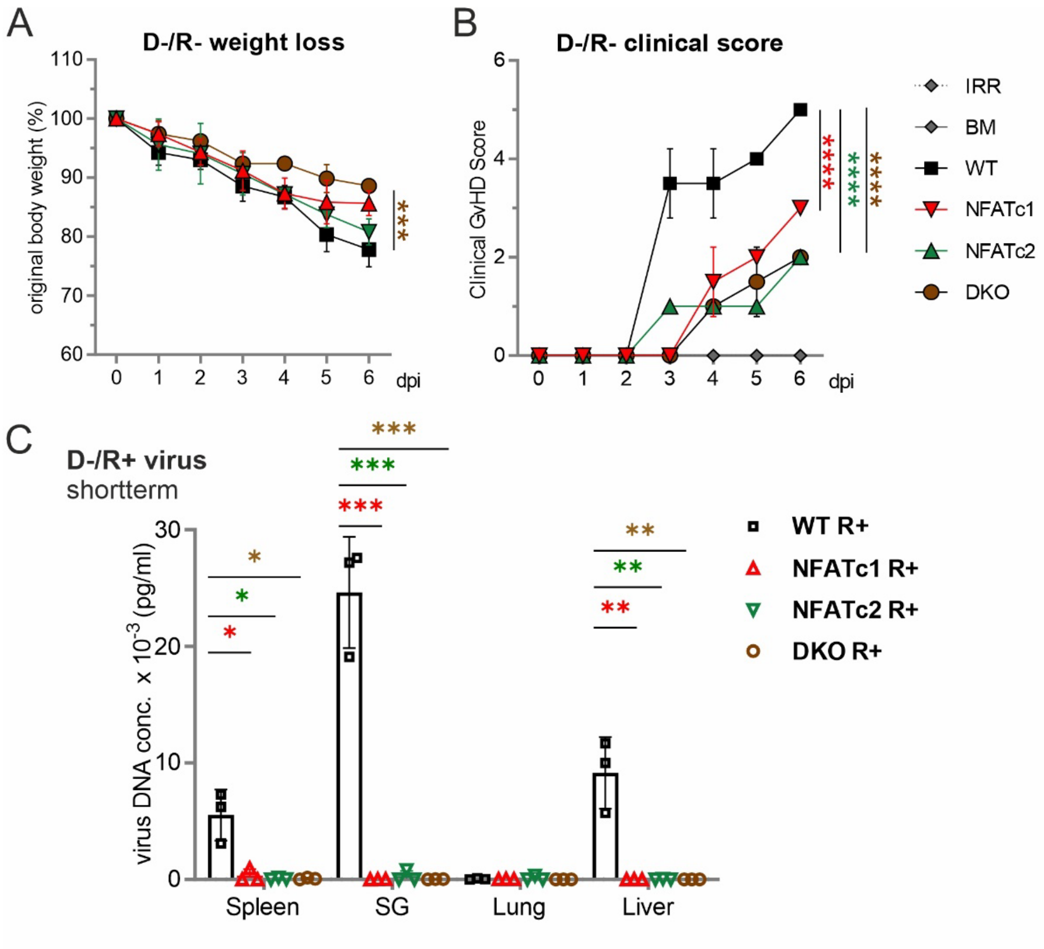

Figure S2. NFAT single or double-deficient T cells ameliorate aGvHD in a parent to F1 setting, related to Figure 2

Acute GvHD was initiated by transferring H-2^{b} donor T cells together with BM cells into H-2^{b+d} CB6F1 recipients. (**A**) Weight loss and (**B**) clinical scores were monitored in non-infected mice for 8 days post allo-HCT. Two-way ANOVA and Tukey's multiple comparisons test (***p < 0.001, ****p<0.0001), mean ± SEM, n≥5, three independent experiments. (**C**) Virus DNA concentration of latently infected mice 8 days after allo-HCT in indicated organs determined by RT-PCR, mean ± SD, n=3, unpaired Student's t test (*p < 0.05, **p < 0.005, ***p < 0.001), two independent experiments.

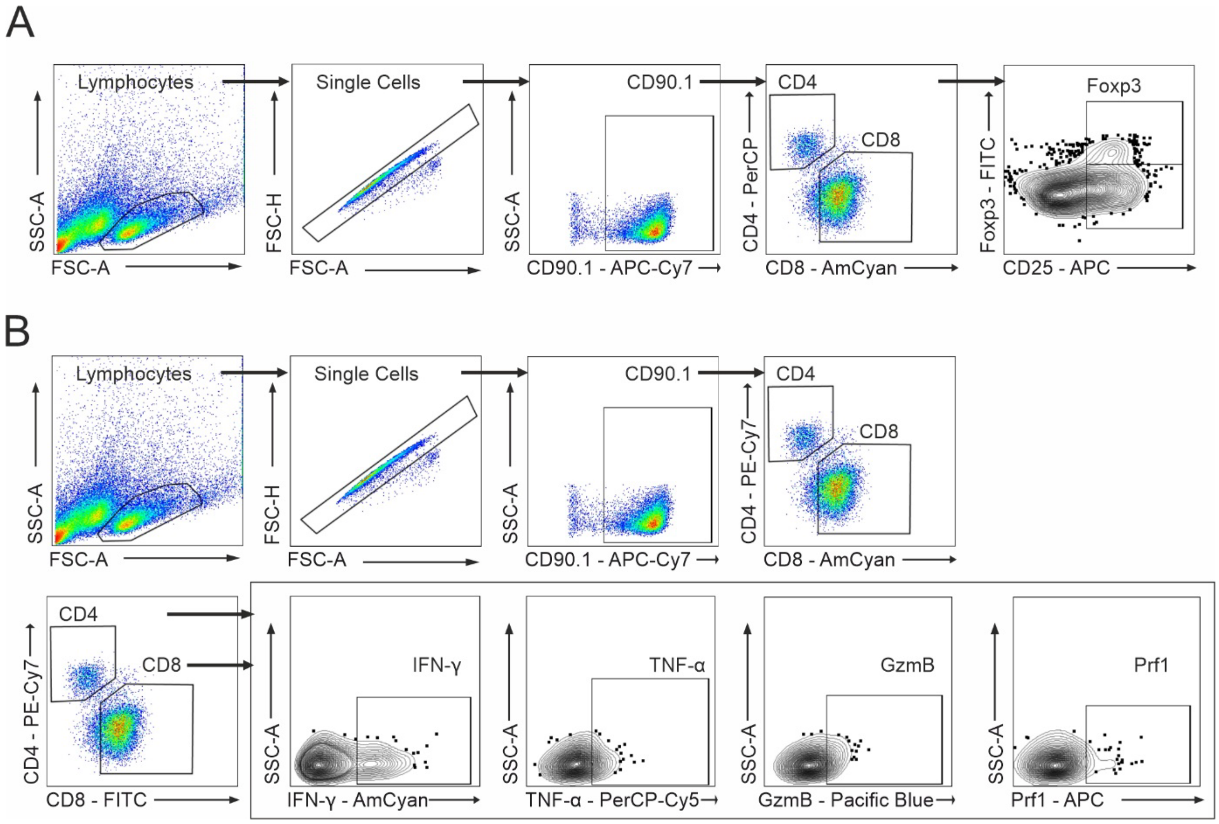

Figure S3. Representative gating strategies, related to Figure 3

(**A**) Gating strategy to identify Tregs within CD90.1$^+$CD4$^+$ T cells by intracellular staining of Foxp3 and surface staining of CD25. (**B**) Gating strategy to determine the expression of cytokines (IFN-γ and TNF-α) and cytotoxic molecules (GzmB and Prf1) within CD4$^+$ and CD8$^+$ T cells.

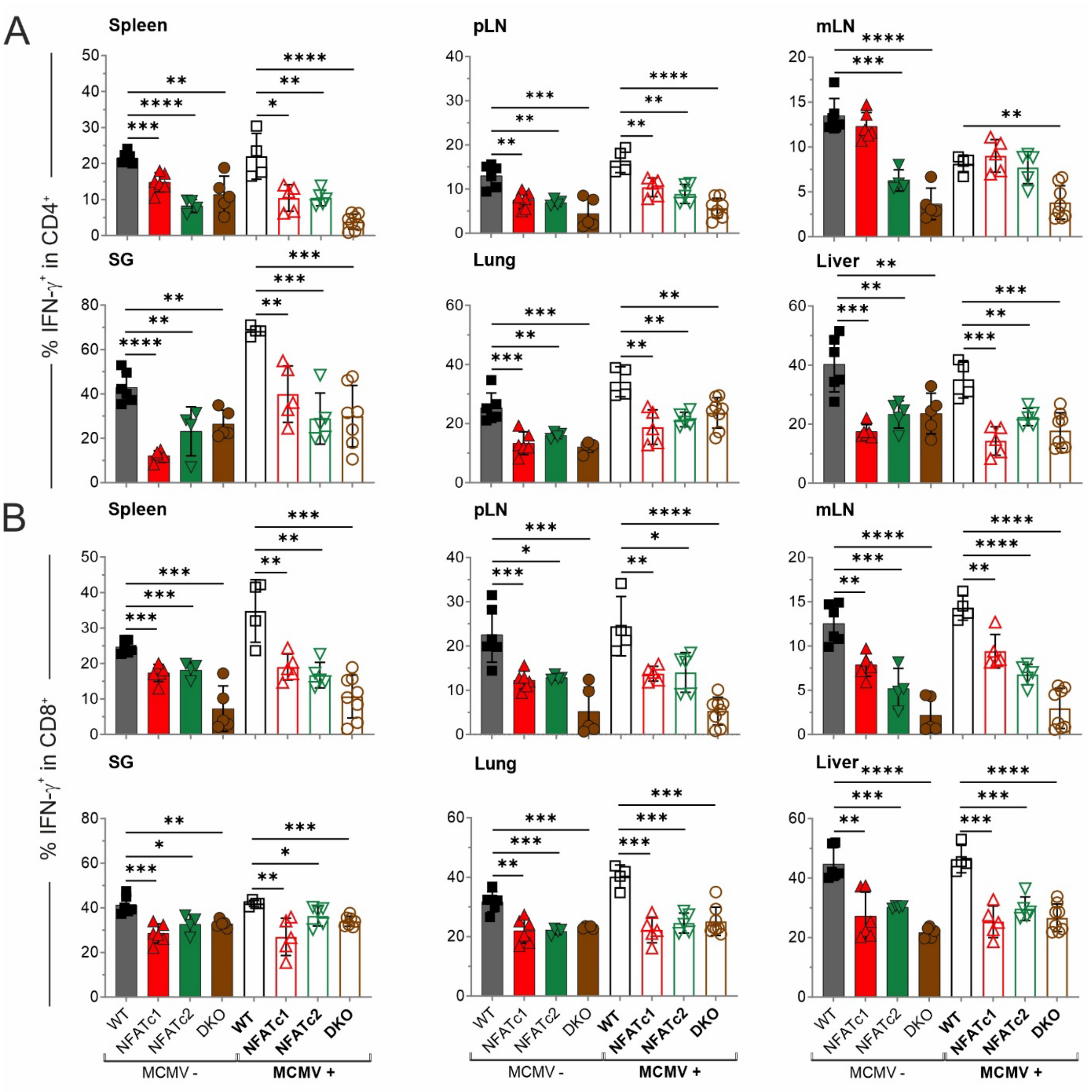

Figure S4. Less CD4⁺ and CD8⁺ T cells express IFN-γ during allo-HCT with or without acute MCMV infection when lacking NFAT, related to Figure 4

(**A**) Frequency of IFN-γ^+ in $CD4^+$ T cells and (**B**) $CD8^+$ T cells of $CD90.1^+$ donor T cells 8 days after transplantation of mice not infected (MCMV-) and acutely infected on day + 2 (MCMV+). Student's two-tailed t-test (*p < 0.05, **p < 0.005, ***p < 0.001, ****p < 0.0001), mean ± SEM, n≥4, two separate experiments.

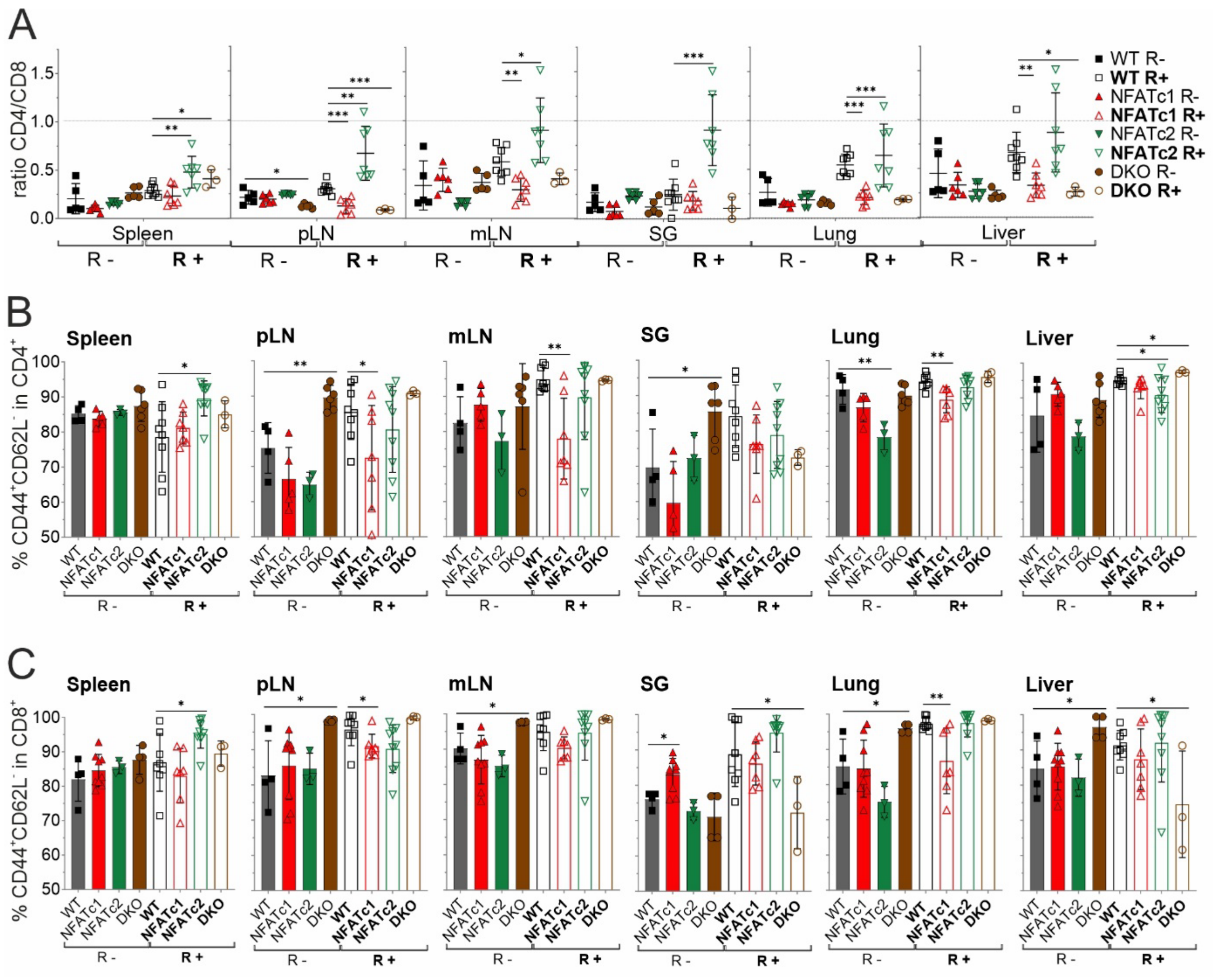

Figure S5. CD4⁺ and CD8⁺ T cells show $T_{EM/EFF}$ phenotype across all conditions, related to Figure 4

(**A**) Ratio of CD4⁺ to CD8⁺ T cells in organs from mice without (MCMV-) and with acute MCMV infection (MCMV+) 6 days post-transplantation. Ratios were calculated based on frequencies. (**B**) Frequency of donor CD90.1⁺CD4⁺CD62L⁻CD44⁺ effector T cells and (**C**) CD90.1⁺CD8⁺CD62L⁻CD44⁺ in spleen, pLN, mLN, salivary glands (SG), lung and liver of latently infected recipient mice (D-/R+) 6 days post transplantation. Student's two-tailed t-test (*p < 0.05, **p < 0.005, ***p < 0.001), mean ± SEM, n≥3, three independent experiments.

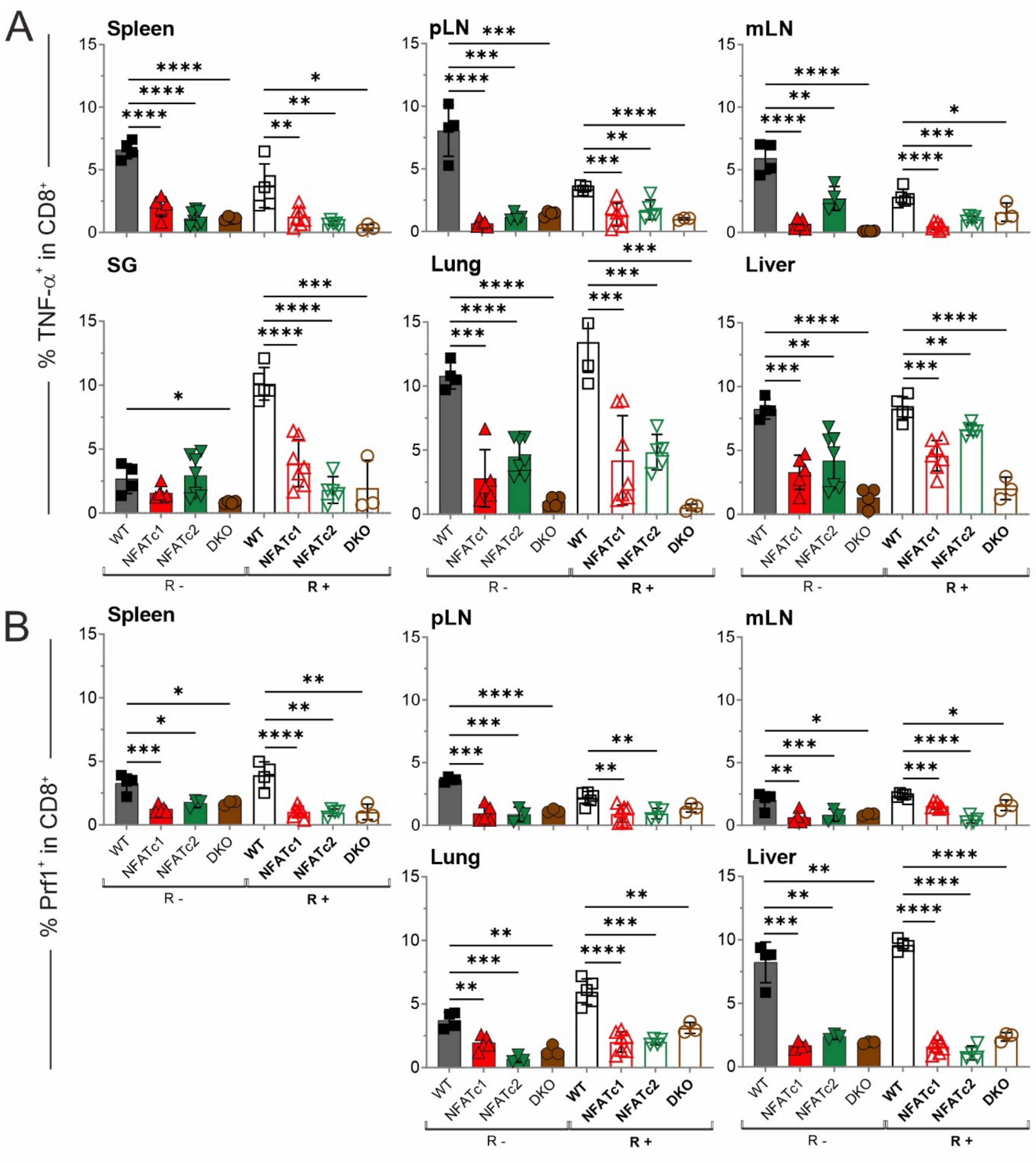

Figure S6. After allo-HCT in latently infected recipients, NFAT-deficient CD8+ T cells are less frequently TNF-α+ or Prf1+, related to Figure 4

The percentage of **(A)** TNF-α and **(B)** Prf1 expression in donor CD8+ T cells in spleen, peripheral lymph nodes (pLN), mesenteric lymph nodes (mLN), salivary glands (SG), lung, and liver of latently infected recipients 6 days post-transplantation. Student's two-tailed t-test (*$p < 0.05$, **$p < 0.005$, ***$p < 0.001$, ****$p < 0.0001$), mean ± SEM, n≥3, two independent experiments.

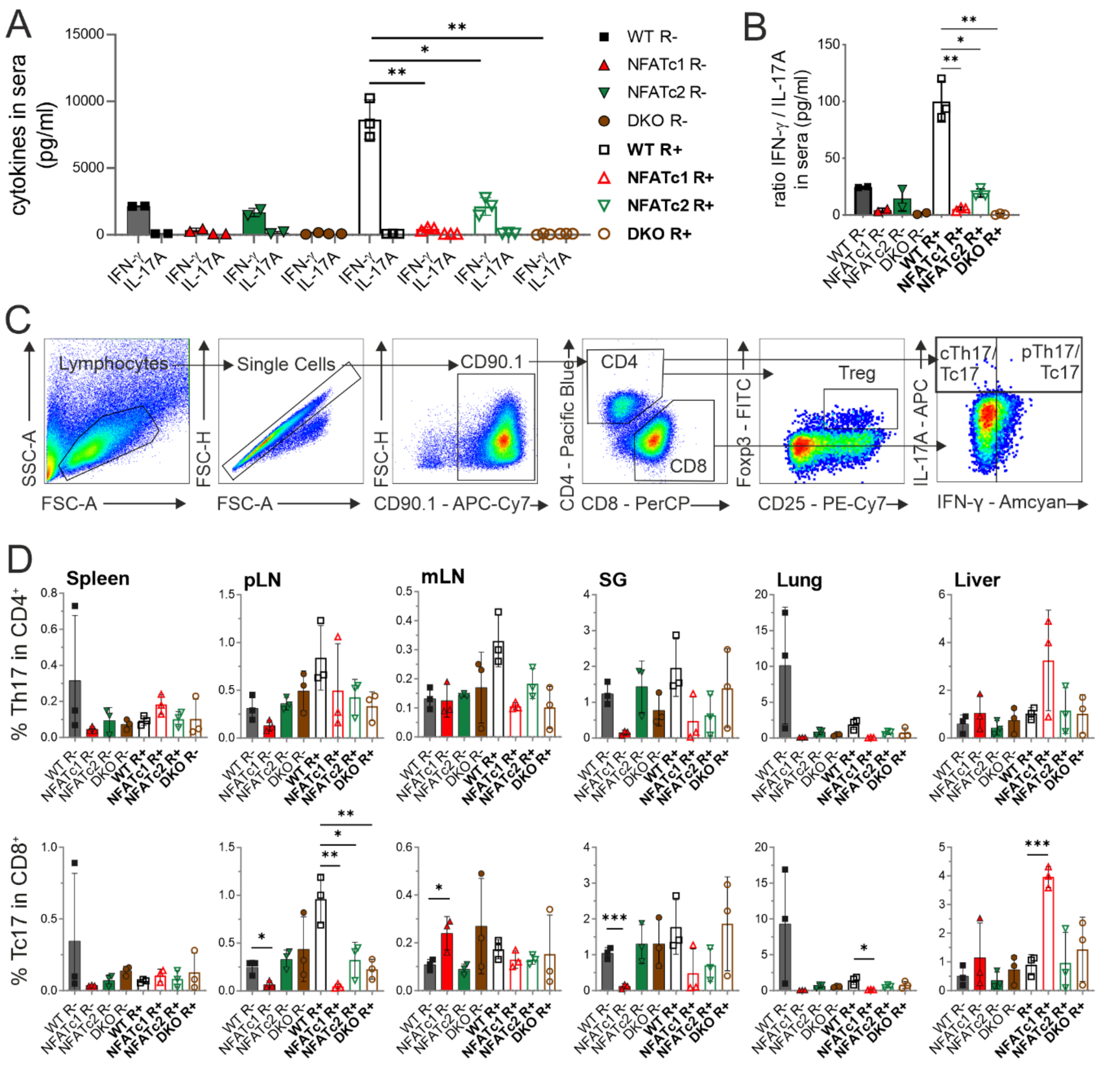

Figure S7. IL-17 is less abundantly secreted than IFN-γ, related to Figure 4

(A) IFN-γ and IL-17A levels (pg/ml) in the sera of aGvHD mice without MCMV infection (R-) and with latent MCMV infection (R+) at 6 days post-transplantation. **(B)** The IFN-γ/IL-17A concentration ratio (pg/ml) in the serum of the same mice as shown in (A). **(C)** Gating strategy to identify Tregs among CD90.1$^+$CD4$^+$ T cells by intracellular Foxp3 staining and surface CD25 staining, and to detect conventional Th17 and Tc17 cells (cTh17/Tc17) and pathogenic IL-17A$^+$IFN-γ$^+$ Th17 and Tc17 cells (pTh17/Tc17) within CD90.1$^+$CD4$^+$ or CD90.1$^+$CD8$^+$ T cell populations. **(D)** Percentages of total Th17 cells within CD4$^+$ T cells and Tc17 cells within CD8$^+$ T cells across the indicated organs. Student's two-tailed t-test (*p < 0.05, **p < 0.005, ***p < 0.001), mean ± SEM, n≥2.

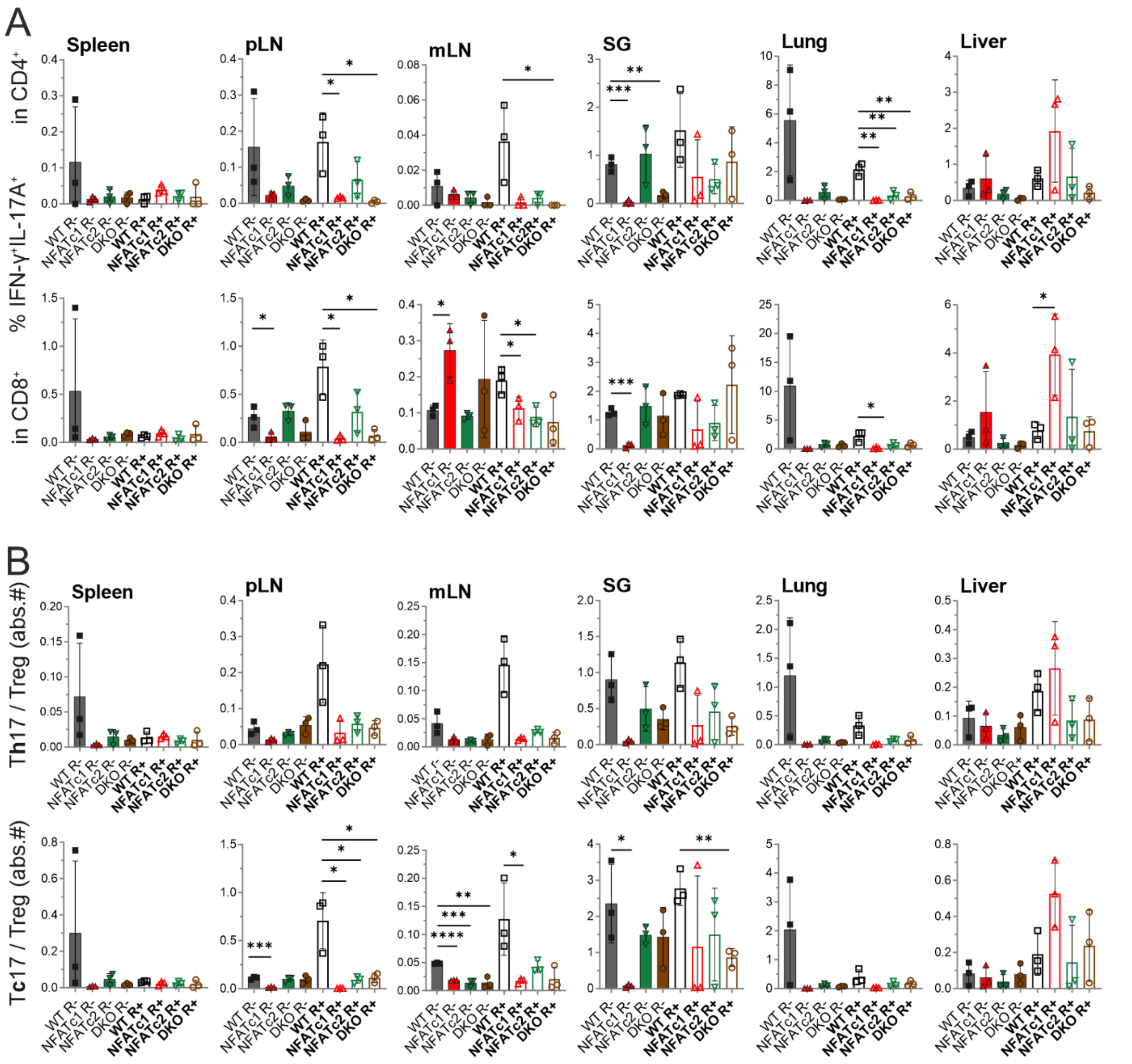

Figure S8. The ratio of the absolute numbers of all Th17 or Tc17 to CD4⁺ Treg cells favors Tregs, related to Figure 4

(A) Percentages of IFN-γ/IL-17A double-producing CD4⁺ Th17 and CD8⁺ Tc17 T cells in the indicated organs of aGvHD mice without MCMV infection (R-) or with latent MCMV infection (R+) at 6 days post-transplantation. **(B)** Ratios of absolute numbers of Th17/Tregs and Tc17/Tregs in different organs of the same mice shown in (A). Student's two-tailed t-test (*p < 0.05, **p < 0.005, ***p < 0.001, ****p < 0.0001), mean ± SEM, n=3.

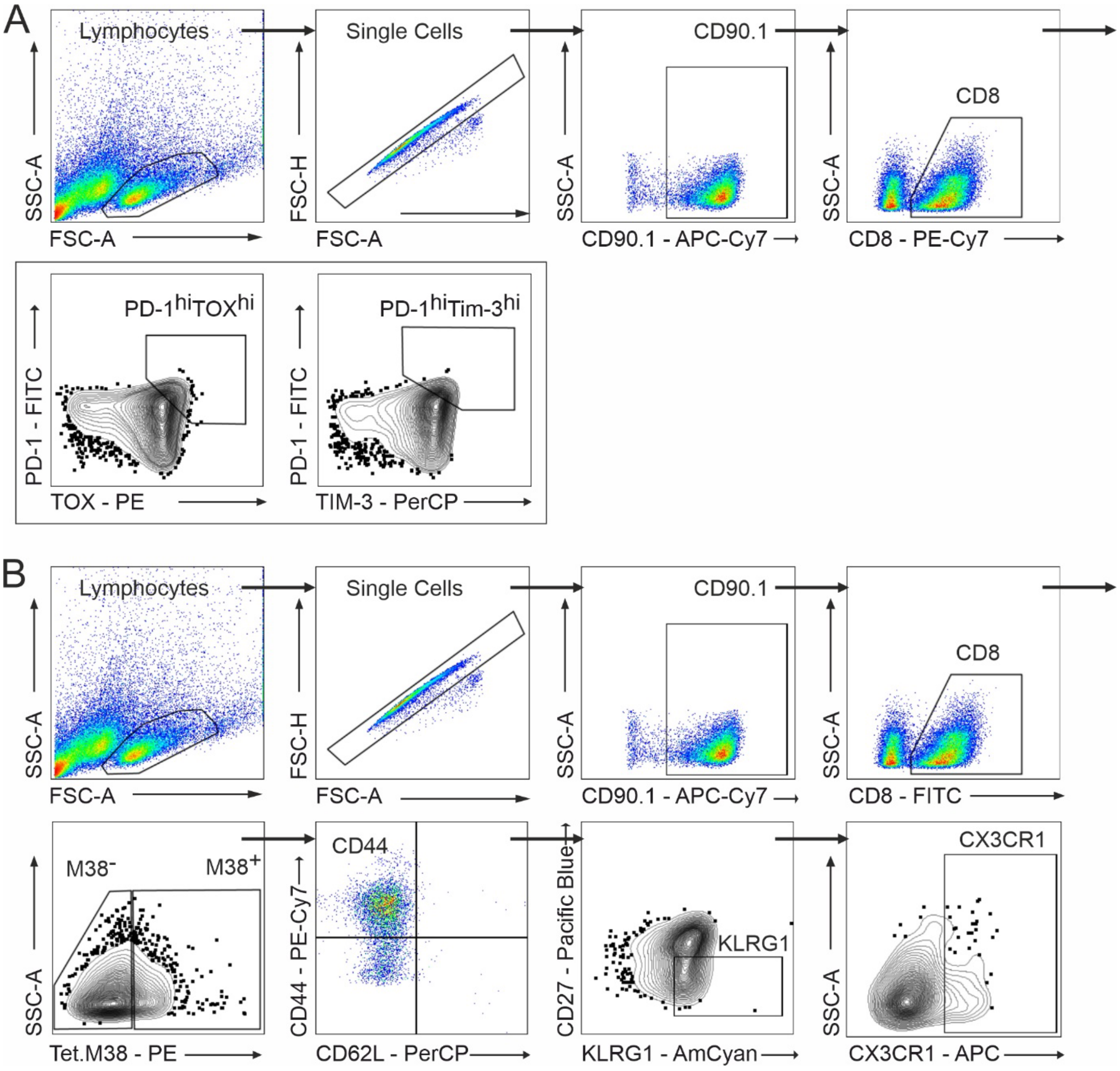

Figure S9. Gating strategy for exhaustion and memory inflation, related to Figure 5

(**A**) Assessment of surface PD-1 on CD8⁺ T cells, followed by intracellular staining of TOX for T_{EX}. (**B**) Memory inflation was identified utilizing tetramer staining of M38 and anti-CD8, -CD44, -KLRG1, -CD27, and -CX3CR1.

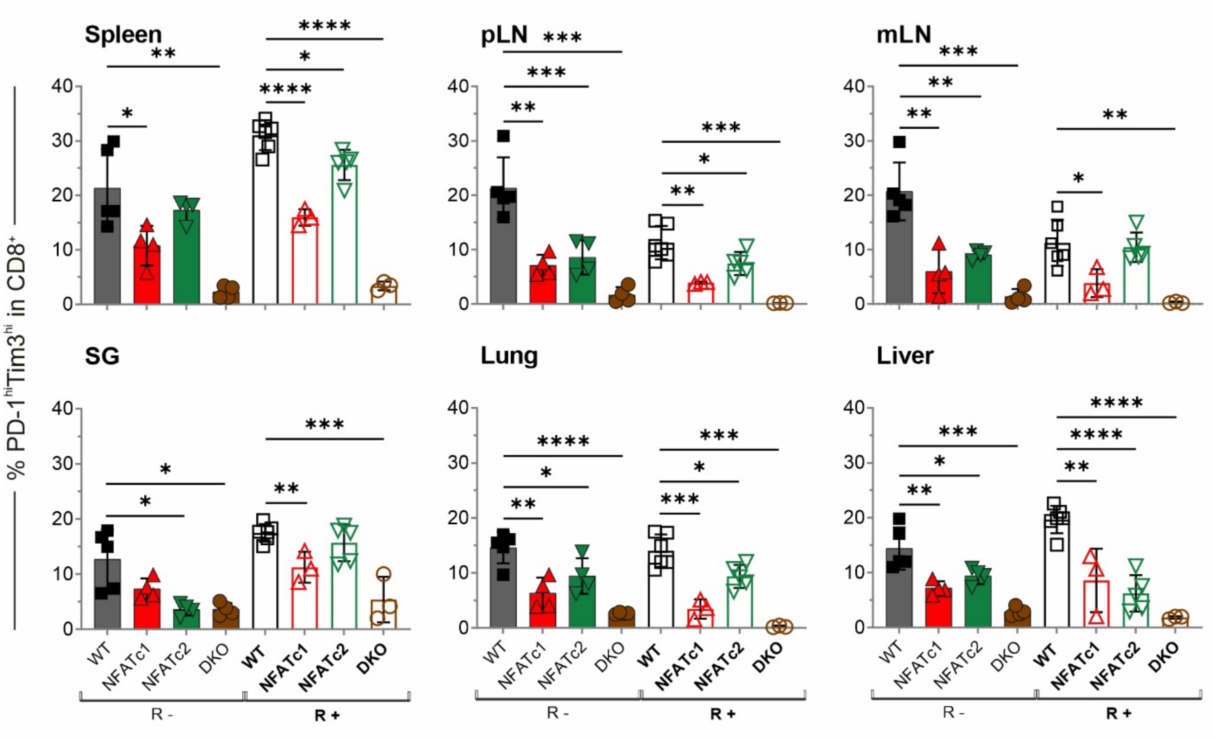

Figure S10. NFAT-deficient CD8⁺ T cells exhibit reduced exhaustion after allo-HCT, related to Figure 5

Frequency of donor CD90.1⁺CD8⁺PD-1ʰⁱTim3ʰⁱ T cells was determined in spleen, peripheral lymph nodes (pLN), mesenteric lymph nodes (mLN), salivary glands (SG), lung, and liver 6 days post-transplantation. Student's two-tailed t-test (*p < 0.05, **p < 0.005, ***p < 0.001, ****p < 0.0001), mean ± SEM, with n≥3, two independent experiments.

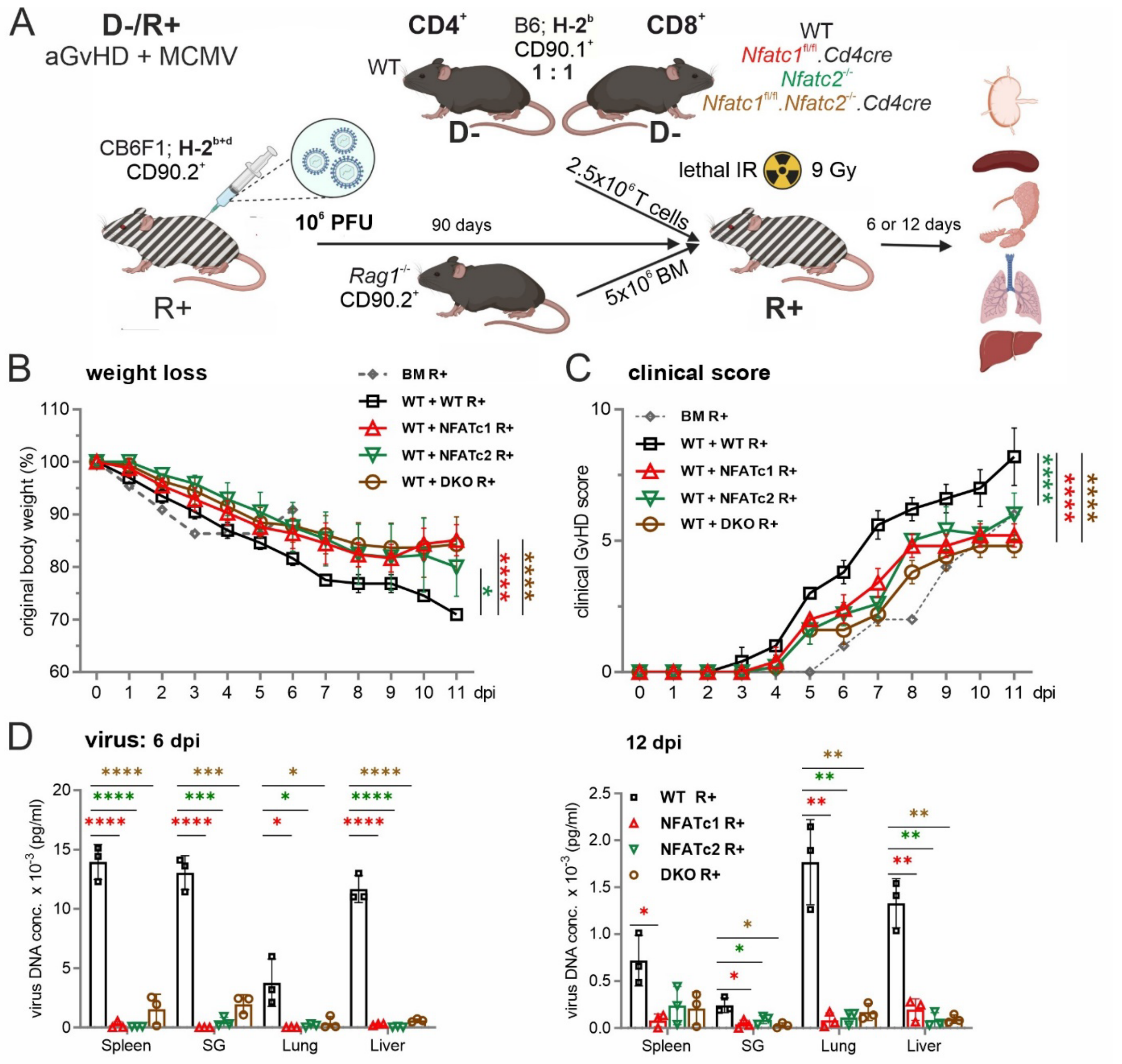

Figure S11. NFAT deficiency in CD8⁺ T cells alone improves GvHD and viral control, related to Figure 6

(**A**) Experimental *in vivo* set up. WT-MCMV Smith strain-latently infected CB6F1 recipients (90 days prior transplantation) were transplanted in a 1:1 ratio with WT $CD4^+$ T cells and $CD8^+$ T cells of distinct genotypes (*Nfatc1*$^{fl/fl}$*.Cd4cre*, *Nfatc2*$^{-/-}$*.Cd4cre*, and *Nfatc1*$^{fl/fl}$+*Nfatc2*$^{-/-}$*.Cd4cre*) expressing CD90.1. Acute GvHD was initiated by transplantation of H-2^b donor T cells together with BM cells into H-2^{b+d} lethally irradiated (9 Gy) CB6F1 recipients. 6- or 12-days post-transplantation, *ex vivo* analyses were conducted. (**B**) Weight loss and (**C**) clinical scores were determined over 12 days. Two-way ANOVA and Tukey's multiple comparisons test (*$p < 0.05$, ****$p < 0.0001$), mean ± SEM, n≥5. (**D**) Virus DNA concentration in indicated organs in short- (6 dpi) and long-term (12 dpi) were determined by RT-PCR, mean ± SD, n=3. (D+F) unpaired Student's t test (*$p<0.05$, **$p<0.005$, ***$p<0.001$, ****$p<0.0001$), two independent experiments.

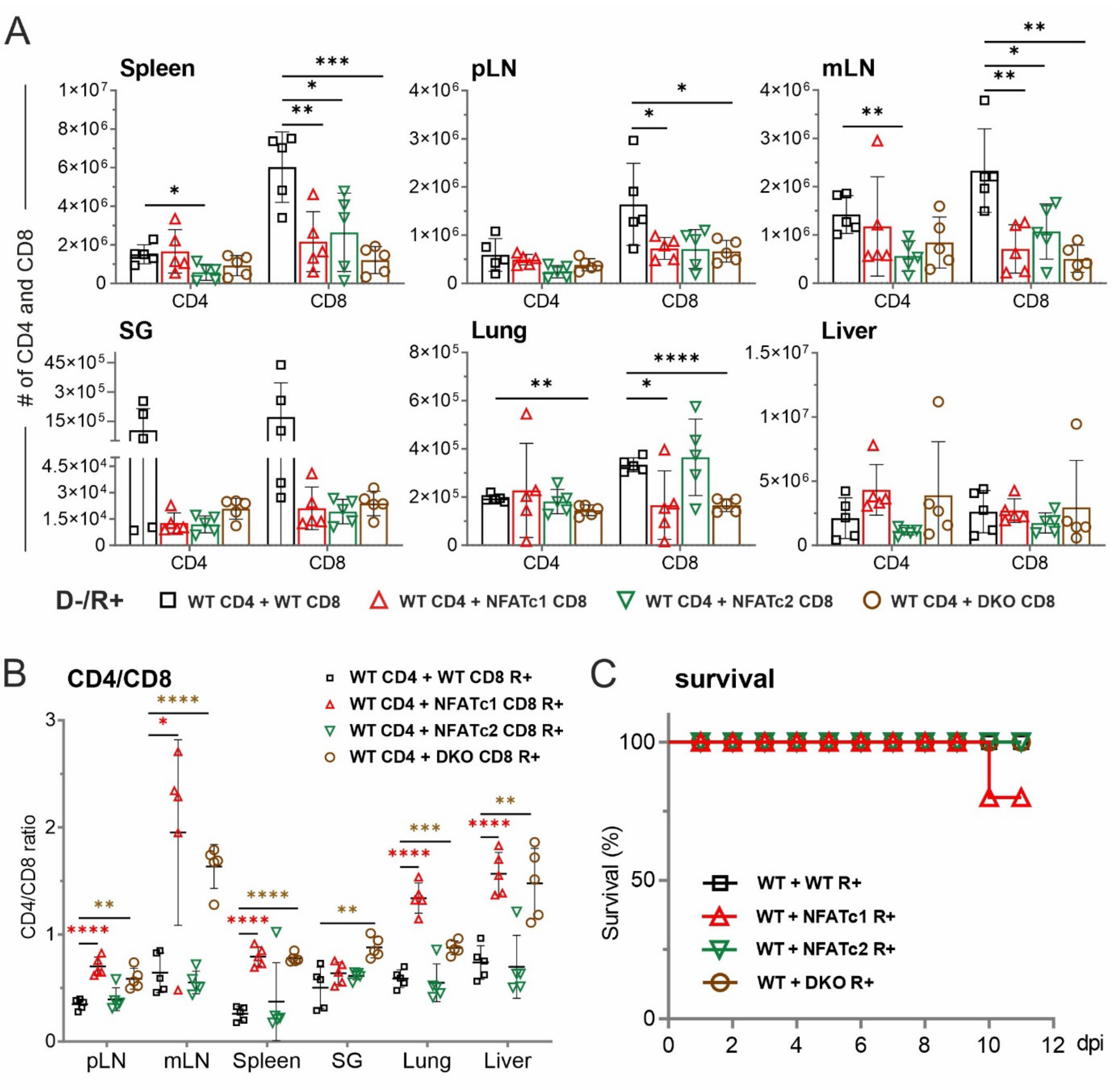

Figure S12. NFAT-deficient CD8⁺ T cells expand less than WT CD8⁺ T cells in the presence of largely unaffected WT CD4⁺ T cell numbers, related to Figure 6

(**A**) Absolute numbers of CD4⁺ and CD8⁺ T cells were determined in indicated organs of latently MCMV-infected recipients at 6 days post transplantation. Student's two-tailed t-test (*p < 0.05, **p < 0.005, ***p < 0.001, ****p < 0.0001), mean ± SEM, n=5. (**B**) Ratio of CD4 to CD8 was calculated based on percentages. (**C**) Survival was monitored daily for 12 days.

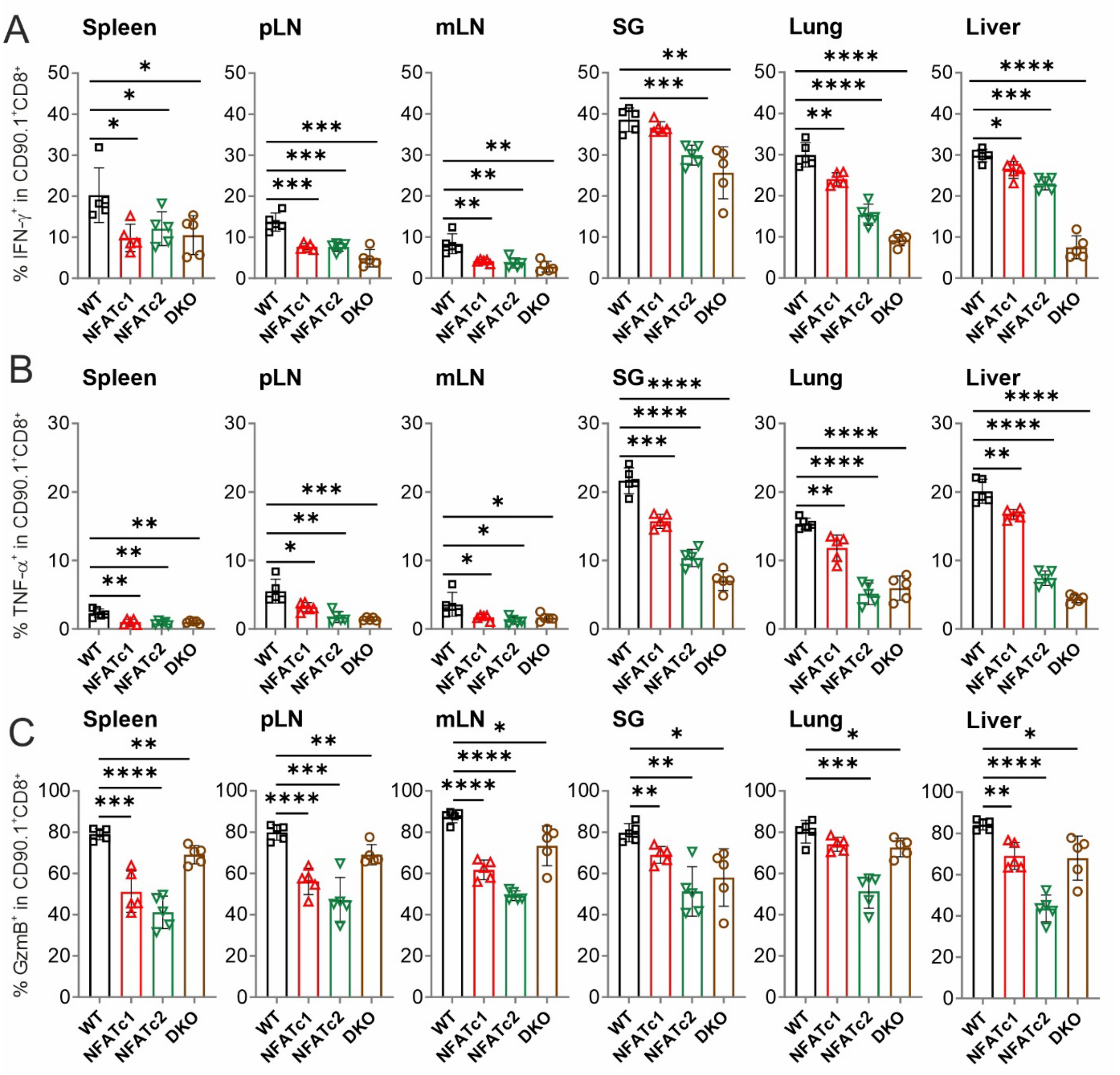

Figure S13. Despite the presence of WT CD4⁺ T cells, NFAT-ablated CD8⁺ T cells present less as IFN-γ⁺, TNF-α⁺, and GzmB⁺, related to Figure 6

The frequency of donor-derived **(A)** CD90.1⁺CD8⁺IFN-γ⁺ **(B)** CD90.1⁺CD8⁺TNF-α⁺ and (**C**) CD90.1⁺CD8⁺GzmB⁺ T cells was assessed using intracellular staining and flow cytometry analysis of the indicated organs at 6 days post-transplantation of 1:1 WT CD4⁺ and WT or NFAT-deficient CD8⁺ T cells. Student's two-tailed t-test (*p < 0.05, **p < 0.005, ***p < 0.001, ****p < 0.0001), mean ± SEM, n=5.

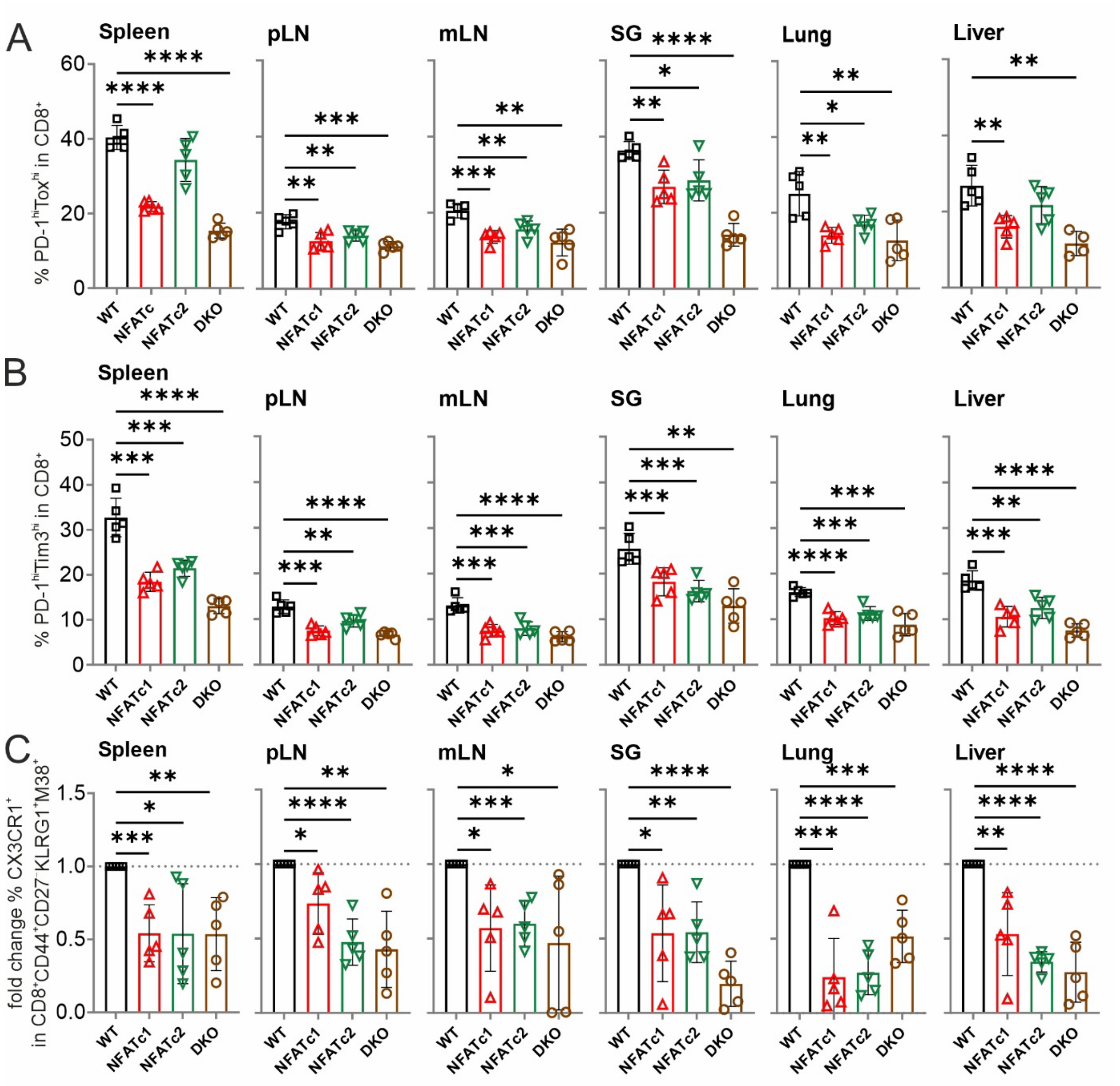

Figure S14. Lack of NFAT exclusively in CD8⁺ T cells results in decreased T_{EX} and CX3CR1⁺ MCMV-specific T cells, related to Figure 6

WT CD90.1$^+$CD4$^+$ T cells and CD90.1$^+$CD8$^+$ T cells isolated from WT, *Nfatc1*$^{fl/fl}$.*Cd4cre*, *Nfatc2*$^{-/-}$.*Cd4cre*, and *Nfatc1*$^{fl/fl}$+*Nfatc2*$^{-/-}$.*Cd4cre* were allo-transplanted (H-2^b→H-2^{b+d}). (**A**) Percentage of donor-derived CD90.1$^+$CD8$^+$PD-1^{hi}TOX^{hi} and (**B**) CD90.1$^+$CD8$^+$PD-1^{hi}Tim3^{hi} T_{EX} cells was determined 6 days post transplantation. (**C**) Fold change of CX3CR1$^+$ MCMV-specific donor-derived CD90.1$^+$CD8$^+$CD44$^+$CD27$^-$KLRG1$^+$M38$^+$ T cells were quantified by tetramer staining followed by flow cytometry analysis. Student's two- tailed t-test (*$p < 0.05$, **$p < 0.005$, ***$p < 0.001$, ****$p < 0.0001$), mean ± SEM, n=5.

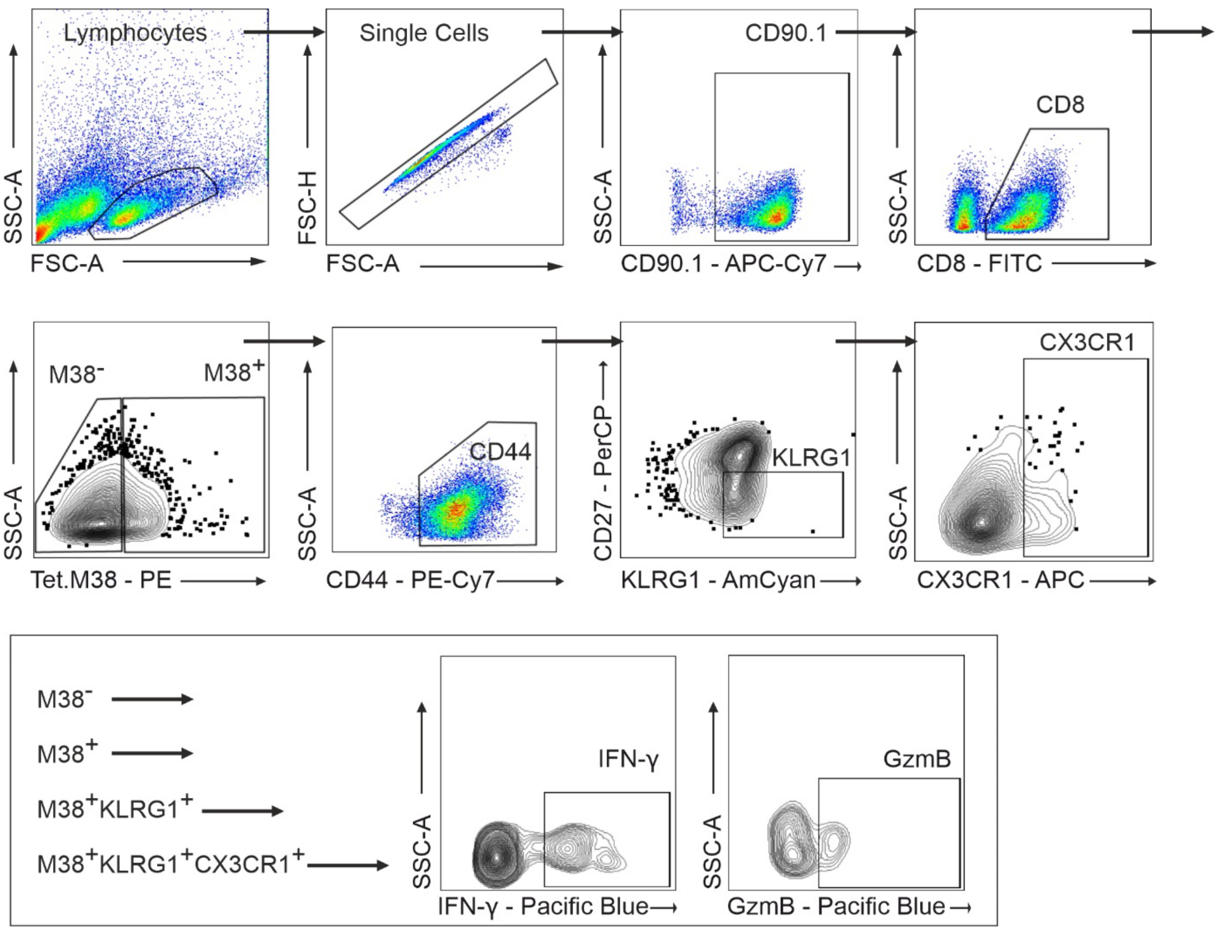

Figure S15. Gating strategy employed for assessing MCMV-specific CD8$^+$ T cells, related to Figure 7

(**A**) A detailed gating strategy was applied to identify MCMV-specific CD8$^+$ T cells, utilizing tetramer staining for M38 and the surface markers CD8, CD44, KLRG1, CD27, and CX3CR1. Intracellular staining was performed to detect the proinflammatory cytokine IFN-γ and the cytotoxic molecule GzmB within M38$^-$ *vs* M38$^+$, M38$^+$CD44$^+$KLRG1$^+$, and M38$^+$CD44$^+$KLRG1$^+$CX3CR1$^+$ cells.

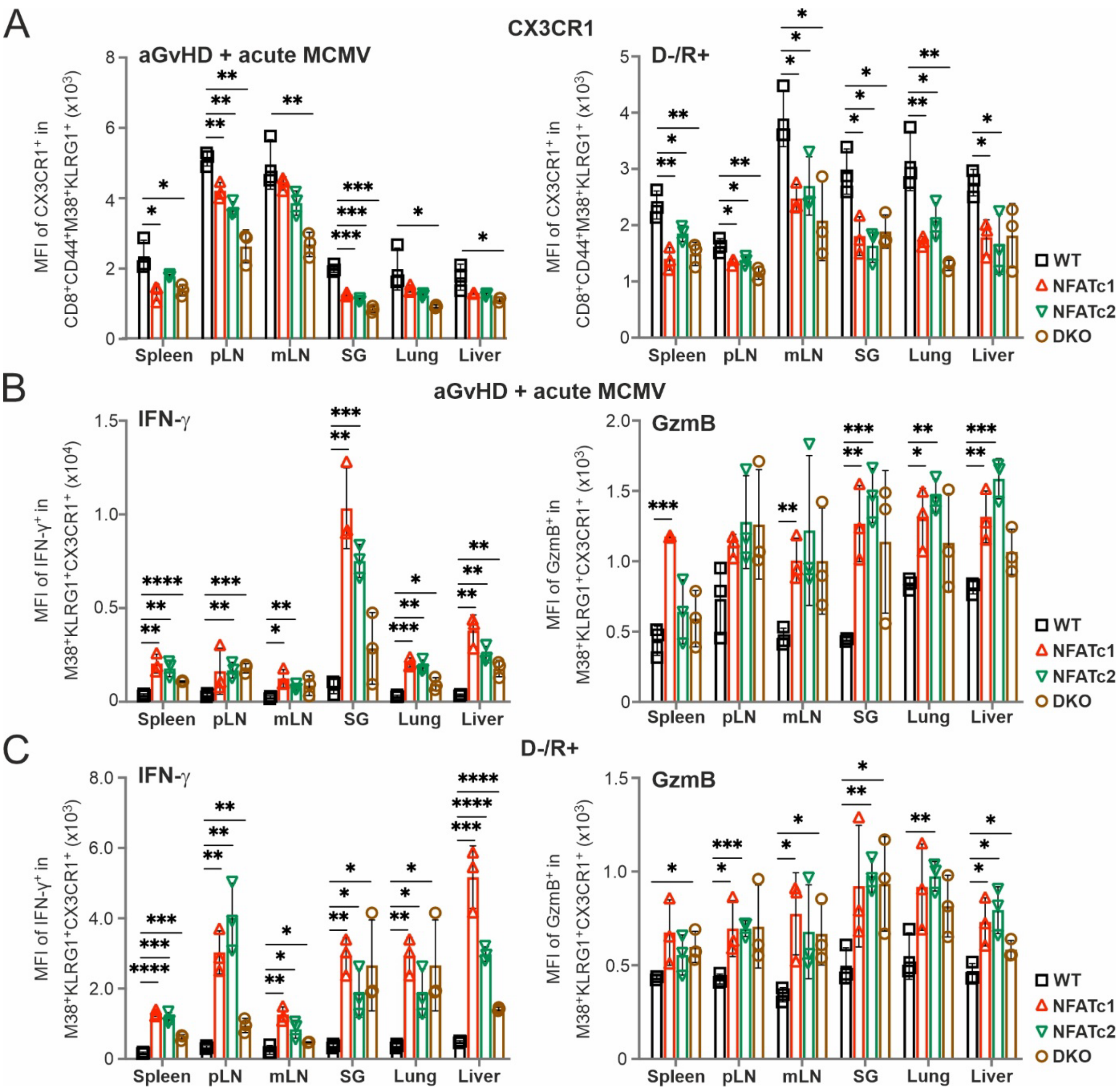

Figure S16. After allo-HCT and acute MCMV infection or in latently MCMV-infected mice, NFAT-deficient MI-T cells present with decreased levels of CX3CR1, but increased levels of IFN-γ and GzmB, related to Figure 7

Mean fluorescence intensity (MFI) of **(A)** CX3CR1 in M38$^+$CD44$^+$CD27$^-$KLRG1$^+$ CD8$^+$ T cells after six or eight days post transplantation. **(B+C)** MFI of IFN-γ and GzmB in M38$^+$CD44$^+$CD27$^-$KLRG1$^+$CX3CR1$^+$ CD8$^+$ T cells after 8 days post-transplantation and acute infection (B) or after 6 days post-transplantation in latently infected mice (C). Cells were restimulated by PMA/Ionomycin *in vitro*. Student's two-tailed t-test (*$p < 0.05$, **$p < 0.005$, ***$p < 0.001$, ****$p<0.0001$), mean ± SEM, n=3.

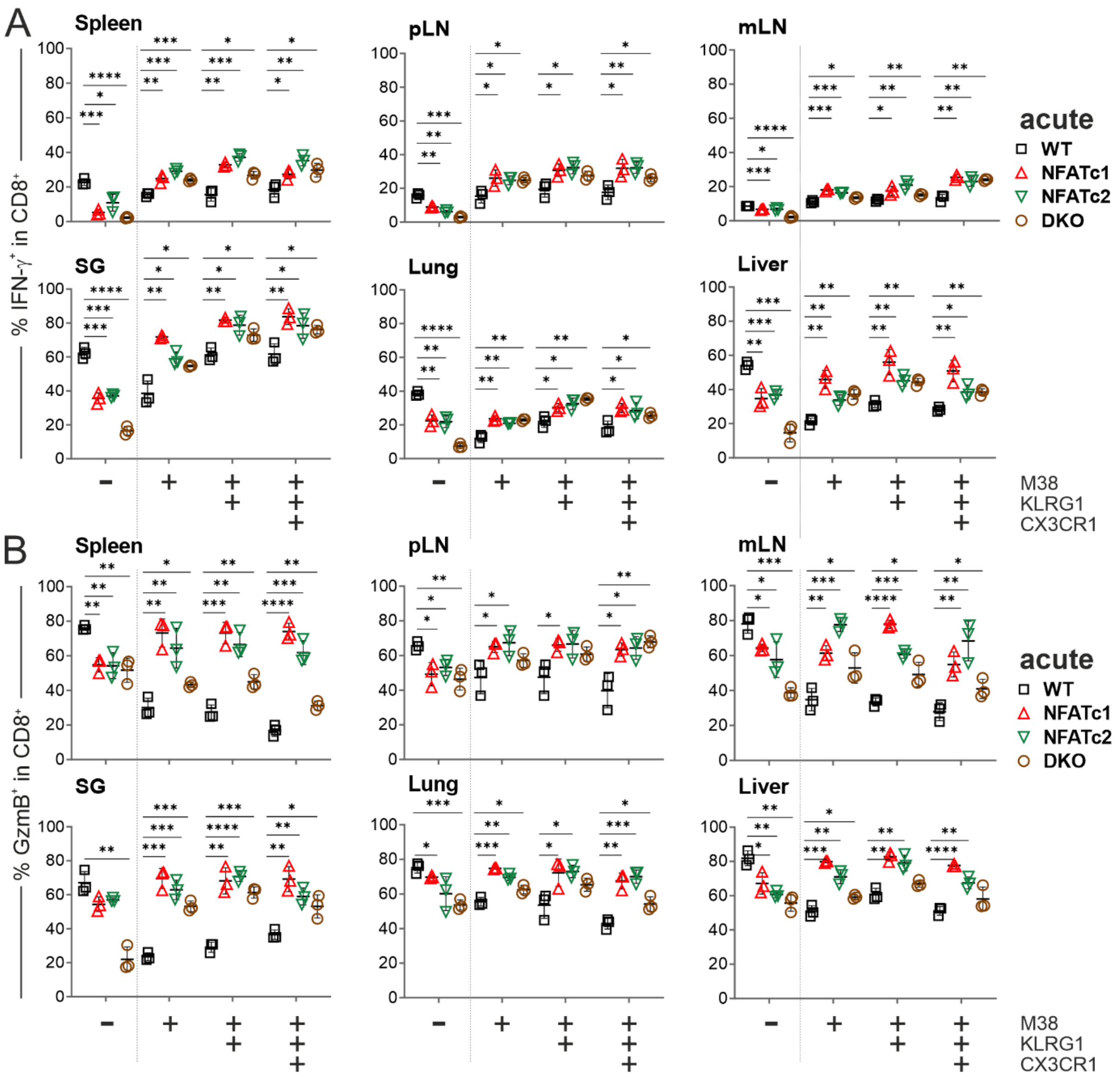

Figure S17. After allo-HCT and acute MCMV infection, NFAT-deficient MI-T cells present with a higher frequency of IFN-γ^+ and GzmB$^+$ cells, related to Figure 7

Percentage of **(A)** IFN-γ^+ and **(B)** GzmB$^+$ cells of either M38$^-$ or MCMV-specific M38$^+$, M38$^+$KLRG1$^+$, and M38$^+$KLRG1$^+$CX3CR1$^+$ CD8$^+$ T cells after 8 days post-transplantation and *in vitro* restimulation. Student's two-tailed t-test (*p < 0.05, **p < 0.005, ***p < 0.001, ****p<0.0001), mean ± SEM, n=3.
